# How is Gaia doing? Trends in global land degradation and improvement

**DOI:** 10.1007/s13280-025-02179-9

**Published:** 2025-04-24

**Authors:** Zhanguo Bai, Jason Daniel Russ, Kentaro Florian Mayr, David Dent

**Affiliations:** 1 ISRIC—World Soil Information, Box 353, 6700 AJ Wageningen, The Netherlands; 2 The World Bank Group, 1818 H Street, NW Washington, DC 20433-0002 USA; 3 Chestnut Tree Farm, Forncett End, Norfolk, NR161HT England, UK

**Keywords:** Carbon capture, Land degradation & improvement, NDVI, NPP, Sustainability

## Abstract

Land degradation is holding back attainment of the Sustainable Development Goals and exacerbating global heating. Land degradation and improvement since 1981 have been assessed by proxy using remotely sensed Normalised Difference Vegetation Index (NDVI), translated into net primary productivity. The quantitative data show the extent and trends of changes over four decades and identify where further action is needed. During 1981–2021, 28.5% of land was degrading—most notably through megafires in boreal forests, land clearance and cultivation in sub-Saharan Africa and the East Indies and across the steppes. The degraded area increased by 4.5% compared to 1981–2003 yet affected fewer people—1.2 billion compared to 1.5 billion. Consistent policies on sustainability increased biological productivity on 26% of land (10.5% more than 1981–2003), particularly cropland in China, India and the European Union; 2.9 billion people now live in the improved areas compared with 0.8 billion in the improving areas of 1981–2003.

**Supplementary Information:**

The online version contains supplementary material available at 10.1007/s13280-025-02179-9.

## Introduction

Land degradation encompasses long-term decline of productivity, environmental integrity, or social value (Folke et al. 2002; Millennium Ecosystem Assessment 2005; Bai et al. 2008; Shukla et al. 2019; Grinand et al. 2020). Most of this degradation is man-made, including impoverishment of vegetation and soil erosion that diminish infiltration of rainfall and recharge of springs and groundwaters; also, nutrient depletion, salinity, sodicity and/or acidity. On a grand scale, we have seen the transformation of the Aral Sea to Aralkum Desert (Gafurova and Juliev 2021), the same actions playing out in the Murray-Darling Basin (Beasley 2021), and continuation of the Dust Bowl of the 1930s across drylands on every continent. Away from the headlines, farm by farm, year on year, these processes are driving millions of people who cannot make a living on their land to leave it—and challenge the frontiers and politics of the wealthy world. This is a global issue (McCammon 1992; Amundson et al. 2015; Borrelli et al. 2017; Olsson et al. 2019; Wang et al. 2023) looming over the Sustainable Development Goals (SDGs), in particular (1) *no poverty*, (2) *zero hunger*, (6) *clean water and sanitation*, (13) *climate action*, and (15) *land degradation neutrality*. Monitoring land degradation and sustainability are both necessary for informed decision-making—and challenging (Tóth et al. 2018; Zhang et al*.* 2018; Prince 2019; Tziolas et al. 2020).

The normalised difference vegetation index (NDVI) recorded by weather satellites measures absorption of photosynthetically active radiation and reflection of unusable heat (Yengoh et al. 2016), thereby capturing photosynthetic capacity and the intensity of life. If more is better, then increased NDVI translated into net primary productivity (NPP) provides an index of land improvement: and a decrease indicates degradation. James Lovelock’s Gaia hypothesis (1979) conceptualises Earth as a self-regulating system where biological, chemical and physical processes interact to maintain conditions favourable for life. Within this frame, NPP plays a crucial role in stabilising atmospheric composition and regulating the carbon cycle. Terrestrial and marine ecosystems constitute planetary-scale feedback by capturing and storing carbon but this activity is disrupted by human-induced land use change and soil degradation. Estimates of annual NPP vary significantly from 53.2 billion or 10^9^ tonnes carbon (GtC) (Melillo et al. (1993) to more recent figures of 112–169 GtC (Sha et al. 2022). Effective management strategies, particularly in agricultural landscapes with big yield gaps or historic soil organic carbon losses, could enhance this natural regulatory capacity with an extra 13.7 GtC (Amelung et al. 2020; Tiefenbacher et al. 2021) and mitigate some of the impacts of anthropogenic CO_2_ emissions, which surpassed 10 GtC in 2021 (United Nations 2024a).

Good-practice guidance for assessing SDG indicator 15.3—the proportion of land that is degraded—considers trends in land cover, land productivity and carbon stocks (Sims et al. 2021). As a proxy for land productivity, we employ the Global Inventory Modelling and Mapping Studies (GIMMS) dataset of remotely sensed NDVI, now comprising 40 years of consistent data. NDVI has been widely applied to assess land degradation (Tucker et al. 1975; Bai et al. 2008; Bai and Dent 2009; Higginbottom and Symeonakis 2014; Yengoh et al. 2016; Kirui et al. 2021; Rivera-Marin et al. 2022; Schillaci et al. 2023), translating into leaf-area index (Myneni et al. 1997), the fraction of photosynthetically active radiation absorbed by vegetation (Asrar et al. 1984), and NPP (Alexandrov and Oikawa 1997). So, we are employing a proxy for a proxy: NDVI for NPP and NPP for land productivity. And declining NDVI or NPP does not necessarily indicate land degradation; nor does increase necessarily indicate improvement. Biological productivity depends on several factors: climate—especially rainfall, sunshine and length of growing season; land use; large-scale ecosystem disturbances such as fires; and the global increase in atmospheric carbon dioxide and active-nitrogen deposition. So, to interpret NDVI and NPP trends in terms of land degradation or improvement, we must eliminate false alarms from climatic variability and land use change. To account for variability of rainfall and, to some extent, soil characteristics, we consider, *rain-use efficiency* (RUE), the ratio of NPP to precipitation (Le Houérou 1984); the combination of remotely sensed NPP and station-observed rainfall has been used to assess land degradation at various scales (Prince et al. 2007; Ibrahim et al. 2015; Chen et al. 2023). We use the same principle to adjust for temperature with *energy-use efficiency* (EUE).

The aim of this study is to identify where sustainability investments and policies may, or may not be, succeeding (at least in terms of biological productivity), and where future efforts should be directed. Understanding land degradation trends is useful for designing effective land management strategies, and by assessing long-term changes in biological productivity, we can judge the success, or otherwise, of current policies. The updated global assessment of land degradation and improvement applies the same procedure as our 1981–2003 assessment (Bai et al 2008; Bai and Dent 2009) which was validated by field observations (Bai et al. 2005; Bai and Dent 2006; Chen and Rao 2008). By drawing on four decades of consistent data, this study supports evidence-based decision-making to achieve land degradation neutrality and other Sustainable Development Goals.

## Materials and methods

**Fig. 1:**
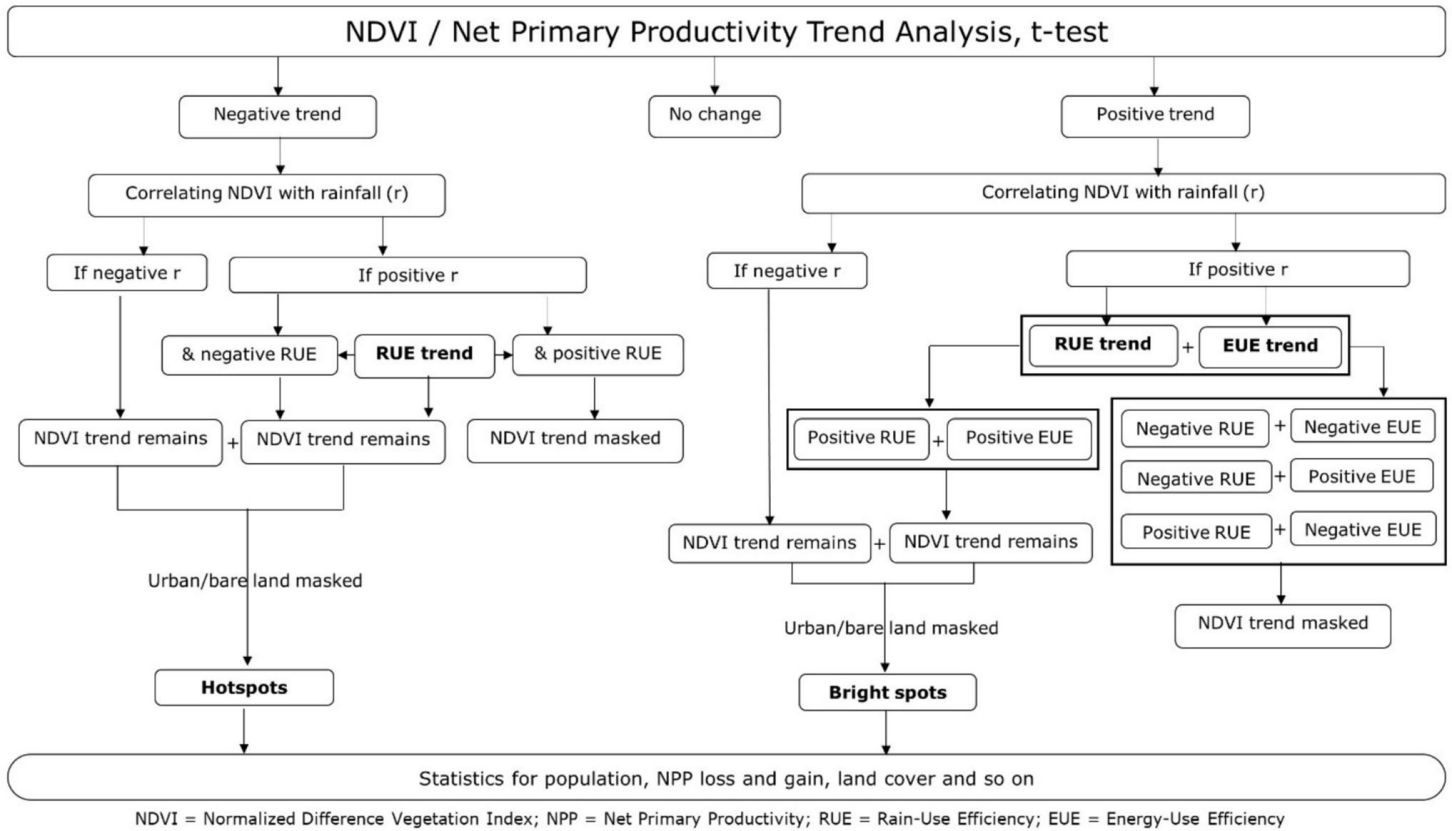
Sequences of data analyses

We derived trends of NPP (carbon capture) at national and global levels from the GIMMS 3rd generation V1.2 (GIMMS–3G +) dataset of global NDVI measurements by the Advanced Very High-Resolution Radiometer (AVHRR) carried on polar-orbiting satellites since 1978. GIMMS compiles corrected and calibrated data from 1982 up to 2021 at a spatial resolution of 0.0833 degree. Maximum NDVI values are composited fortnightly from the sequence of AVHRR sensors, taking account of calibration loss, orbital drift and volcanic eruptions, and provided in Network Common Data format (NetCDF) (Pinzon et al. 2023). Figure 1 summarises our procedure. Some useful terms and definitions appear in Table 1.

**Table 1:** Terms and definitions

Terms	Definitions	Acronyms
Advanced Very High-Resolution Radiometer	Instrument carried by the US National Aeronautics and Space Administration (NASA) weather satellites	AVHRR
Global Inventory Modelling and Mapping Studies	AVHRR dataset maintained by the University of Maryland	GIMMS
Energy-Use Efficiency	Ratio of annual sum NDVI to annual accumulated temperature	EUE
Moderate Resolution Imaging Spectroradiometer	Instrument carried by the NASA Terra and Aqua satellites	MODIS
The Normalised Difference Vegetation Index	Ratio of (Near Infra-red – Red) / (Near Infra-red + Red) spectral bands reflected by vegetation	NDVI
Net Primary Productivity	Energy stored by photosynthesis less energy used for respiration and maintenance per unit area and time	NPP
Rain-Use Efficiency	Ratio of annual sum NDVI to annual rainfall	RUE

***RUE and EUE*** were calculated at the same resolution as GIMMS–3G + data using monthly precipitation and temperature values since January 1981 abstracted from the University of East Anglia Climatic Research Unit Time Series (CRU TS v4.0.6) gridded at 0.5° resolution (Harris et al. 2020).

***MODIS NPP data*** at 500 m resolution (MOD17A3HGF Version 6.1, Running and Zhao 2021) were used to translate GIMMS NDVI data to NPP by re-sampling MODIS data to the same resolution as GIMMS using nearest-neighbour assignment. Correlation between annual sum NDVI and annual NPP at pixel level was calculated for the overlapping period (2000–2021) according to the equation: y = Ax + B, where y is NPP for a given year (kgC/ha/year); x, annual sum NDVI for the given year; A, gradient or slope of the linear regression; B, intercept of the linear regression. NPP loss and gain were calculated by multiplication of multiple-year average of annual NPP (2000–2021) and percentage change of annual sum NDVI from 1981–2021. The percentage change of NDVI was calculated by 100*gradient of annual sum linear trend/multiple-year average of annual sum NDVI 1981–2021.

**Fig. 2:**
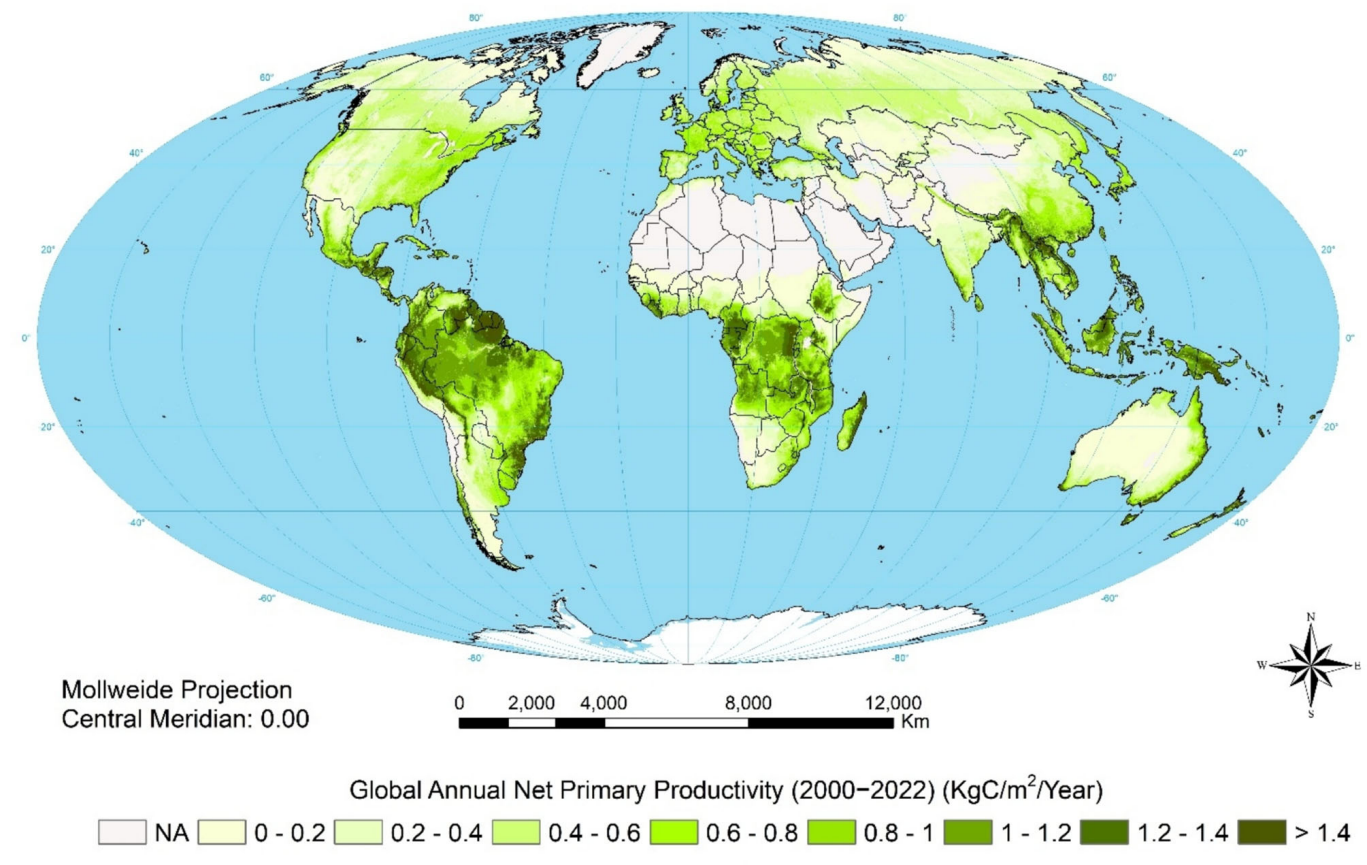
Mean annual NPP 2000–2022, MODIS data from Running and Zhao (2021)

MODIS global NPP data for 2000–2022 were used in Fig. 2 to provide context for the changes revealed over the longer term by the GIMMS data.

***Land cover*** The Copernicus Climate Change Service provides global land cover maps from 2016 to the present at 300 m resolution consistent with the global annual land cover maps from 1992–2015 produced by the European Space Agency Climate Change Initiative land cover project (ESA Copernicus 2023). Land cover 2020 was used to compare with identified land degradation and improvement and to mask urban areas and bare ground.

***Population*** The *Gridded Population of the World, version 4* (CIESIN 2018) provides UN World Population Projection-adjusted population count and population density at various resolutions. The gridded population counts for 2005 and 2020 at 2.5 arc-minutes resolution were compared with indices of land degradation and improvement.

***Trend analysis*** Trends were determined by linear regression. The absolute change (∆) is the gradient of the regression; relevant or percentage change (%) is 100*(the absolute change/multiple-year average of annual sum NDVI 1981–2021). Data were tested for temporal and spatial independence following Livezy and Chen (1983). When the absolute values of the autocorrelation coefficients of lag-1 to lag-3, calculated for a time series consisting of *n* observations, are not larger than the typical critical value, *i.e.* 1.96/$\sqrt n$ corresponding to the 5% significance level, the observations in this time series can be accepted as being independent. The T–test was used to arrange the slope values in classes showing strong or weak positive or negative trends:

$$T = b/se\left( b \right)$$

where *b* is the estimated slope of the regression line between the observation values and time and *se(b)* represents the standard error of *b.*

R language (R Core Team 2023) was used for data processing, trend and correlation analysis; ESRI ArcGIS was used for statistics and map visualisation.

***Degrading areas*** were identified, first, by a negative trend of sum NDVI. To distinguish between productivity decline caused by land degradation and declining productivity due to other factors, rainfall variability and irrigation have been accounted for by:

1. Identifying pixels with a positive relationship between NDVI and rainfall
2. For those pixels, RUE has been considered: where productivity declined but RUE increased, the decline of productivity was attributed to declining rainfall; those areas were masked (urban areas and bare ground were also masked)
3. NDVI trends have been calculated for the remaining areas as *RUE-adjusted NDVI: i.e*., pixels where there was a negative relationship between NDVI and rainfall. NDVI trends were also calculated for pixels with positive relationship between NDVI and rainfall but declining RUE.

***Improving areas*** were identified by: (1) a positive trend in sum NDVI and a negative correlation with rainfall; (2) a positive trend in sum NDVI and positive RUE and EUE. Again, urban areas and bare ground were masked.

***Net primary productivity*** loss in the identified degrading areas or gain in the improving areas was calculated using the translation of NDVI into NPP. The number of people affected was estimated by overlaying the degrading or improving areas with population maps.

## Results and discussion

### Context

**Fig. 3:**
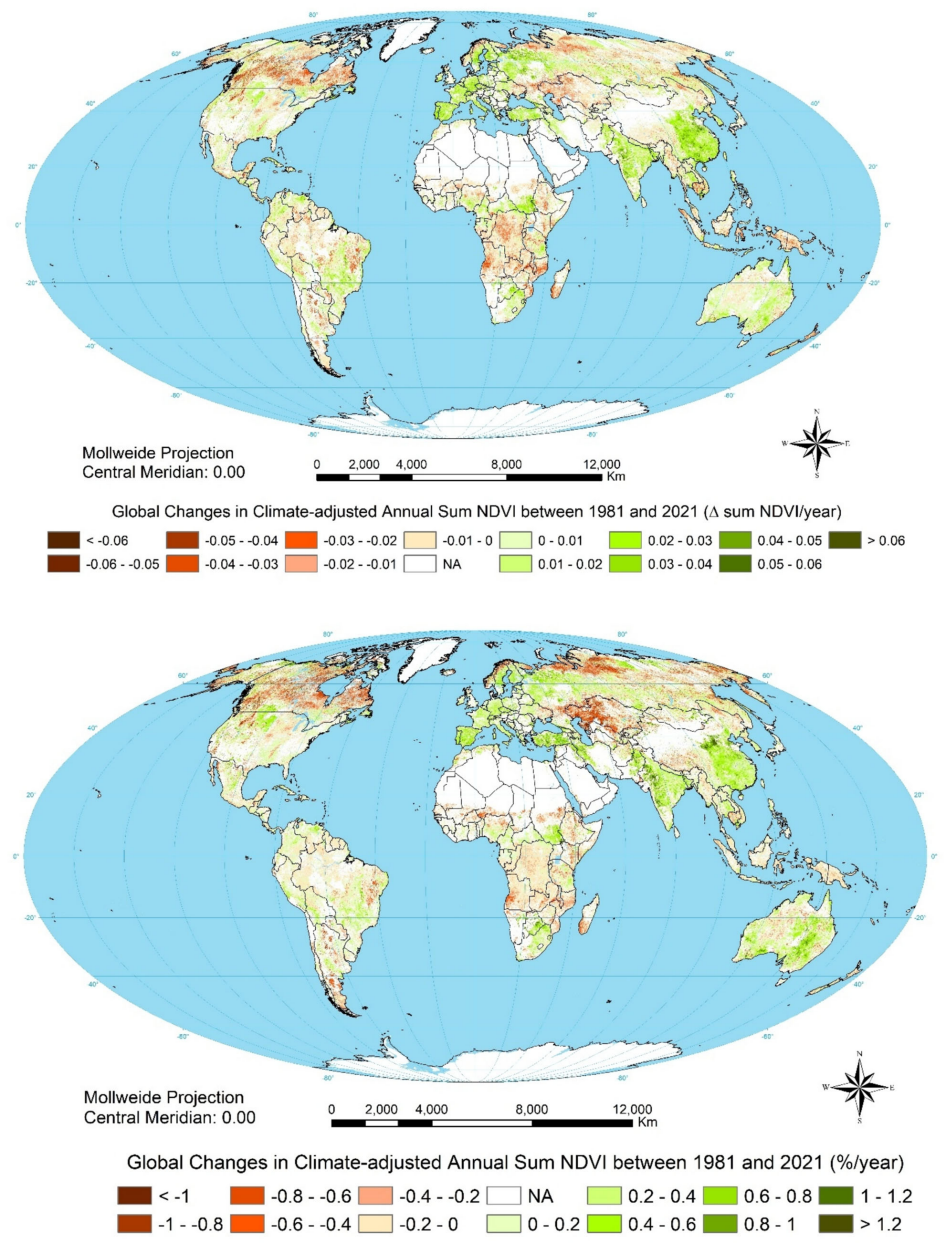
Trends in climate-adjusted annual sum NDVI, 1981–2021: top, absolute; bottom, relative

As context for considering changes in NPP, Fig. 2 shows current global land productivity. Figure 3 then shows climate-adjusted change in NDVI over the period 1981–2021 with urban and barren land masked. This is our measure of land degradation (in red) and improvement (in green). It presents a different picture from earlier assessments that compounded historical land degradation with ongoing changes (Sonneveld and Dent 2007). By focussing on data since 1981, this analysis highlights recent trends but does not capture the extent of past land degradation, much of which is irreversible.

### Caveats concerning global application of the data

1. NDVI performs best as an indicator of NPP in areas with sparse to moderate vegetation cover. Under a dense canopy, additional biomass below the canopy is hidden and its increase brings no detectable change in the signal (Ripple 1985).
2. In compiling the GIMMS database, cloud cover is screened by reading the maximum NDVI values within twice-monthly compositing periods (Pinzon et al. 2023). Even so, prolonged cloud cover may result in an underestimation of NDVI.
3. The spatial variability of rainfall in drylands makes interpolation of point measurements problematic, and meteorological stations are sparse in many of these areas.
4. NDVI is only a proxy. It tells us nothing about the kind of degradation or improvement. What is happening in, say, burning boreal forests is different from what happened in New Zealand when price support for agriculture was abandoned in 1984. However, because the index is mapped as a continuous surface, the drivers may be revealed by correlation with other geo-located biophysical and socio-economic data.
5. As a measure of land degradation or improvement, loss or gain of NPP has been calculated for those areas where *both* NPP and RUE are declining (*i.e.* land degradation) or where NPP, RUE and EUE are increasing (*i.e.* land improvement). This is likely to be a conservative estimate since, globally, NPP has increased over the study period. However, in areas where NPP is increasing while RUE and EUE are declining, some land degradation may already be occurring, even if it is not yet reflected in declining NPP. If we only consider areas where both indices are decreasing, such early-stage degradation would go undetected. For this reason, RUE or EUE should be used alone for early warning of land degradation or a herald of improvement; otherwise, we might overlook promising interventions that increase RUE or EUE but have not yet brought about increasing NPP.
6. Additionally, some degradation or improvement, notably concerning biodiversity and consequently resilience, is not fully captured by NPP.

We acknowledge these caveats and return to them in the Discussion and Conclusions.

### Land degradation

Global analysis reveals that, over the period 1981–2021, 28.5% of land has been degrading—4.5% more than our previous assessment over 1981–2003 (Bai et al. 2008). But 26.2% of land has been improving—10.5% more than earlier. About 1.2 billion people live in the 1981–2021 degrading areas—0.3 billion less than in areas so identified earlier. Considering the global population increased by one quarter over the last 20 years (United Nations 2024b), this might be considered a positive result but can be attributed in large measure to abandonment of the land.

Table 2 lists by country areas of land degradation and improvement, NPP loss and gain, and directly affected people. *Directly affected people* refers to the rural population of the degraded or improved areas although, of course, city dwellers in these catchments are not unaffected, not least those recently relocated from these same areas (Tables 3 and 4).

**Table 2:** Areas of land degradation and improvement, NPP loss and gain, and affected people by country between 1981 and 2021*

Country	Degrading area km^2^	% territory	% global degrading area ktC/41 years	Total NPP loss	LD affected people, millions	% total population in degrading areas	Improving area km^2^
Afghanistan	117 876	18.1	0.27	1763	8.8	21.4	115 033
Albania	1565	5.4	0.004	105	0.03	0.9	12 125
Algeria	49 408	2.1	0.10	883	6.1	13.6	72 409
Andorra	52	11.1	0.00	7	0.01	11.7	364
Angola	835 156	67.0	1.70	44 139	14.0	39.4	88 384
Antigua and Barbuda	0	0.0	0.00	0	0	0.0	220
Argentina	706 032	25.5	1.72	33 512	9.5	20.8	558 455
Armenia	1442	4.8	0.00	28	0.1	4.3	13 252
Australia	1 302 627	17.0	2.87	29 533	1.0	3.8	3 667 202
Austria	5011	6.0	0.02	322	0.1	1.5	23 959
Azerbaijan	16 082	18.6	0.04	652	1.7	16.3	40 306
Bahamas	3020	21.7	0.00	240	0.01	2.8	1975
Bangladesh	7083	4.9	0.01	193	5.0	3.0	110 667
Barbados	72	16.7	0.00	10	0.02	6.0	0
Belarus	12 025	5.8	0.04	405	0.6	6.5	78 854
Belgium	379	1.2	0.00	12	0.07	0.6	24 170
Belize	5372	23.4	0.01	210	0.08	20.4	11 003
Benin	21 917	19.5	0.05	107	2.5	18.7	19 947
Bhutan	12 636	26.9	0.02	514	0.3	32.4	9909
Bolivia	236 303	21.5	0.48	8729	2.2	17.6	424 512
Bosnia and Herzegovina	8379	16.4	0.02	381	0.74	21.8	18 531
Botswana	45 353	7.6	0.09	733	0.1	5.6	180 506
Brazil	2 453 800	28.8	4.97	100 658	31.8	14.8	2 451 035
Brunei	355	6.2	0.00	13	0.002	0.6	1598
Bulgaria	3104	2.8	0.01	85	0.2	2.5	10 261
Burkina Faso	121 213	44.2	0.25	174	9.5	41.9	6473
Burundi	14 010	50.3	0.03	539	6.1	47.5	5869
Cambodia	119 632	66.1	0.24	7551	9.0	53.6	40 212
Cameroon	78 044	16.4	0.15	1745	5.7	20.4	227 221
Canada	4 692 151	47.0	17.4	364 594	7.8	20.4	1 950 420
Cape Verde	1344	33.3	0.00	24	0.07	12.5	896
Central African Republic	68 612	11.0	0.14	2093	0.9	16.4	234 838
Chad	140 009	10.9	0.29	328	6.1	34.3	21 335
Chile	115 417	15.3	0.27	5593	0.9	4.6	100 782
China	1 639 469	17.1	3.93	38 041	118.9	8.3	3 449 516
Colombia	317 253	27.9	0.63	8883	7.3	14.1	491 232
Congo R	199 125	58.2	0.40	5939	2.4	39.9	90 018
Congo DR	1 641 579	70.0	3.21	69 002	56.8	57.4	368 562
Costa Rica	27 926	54.7	0.05	972	2.7	52.4	16 172
Croatia	2142	3.8	0.01	68	0.2	4.2	14 735
Cuba	11 863	10.7	0.03	415	0.9	8.1	53 180
Cyprus	612	6.6	0.00	27	0.2	14.1	4205
Czech Republic	111	0.1	0.00	0.9	0.1	0.9	50 385
Denmark	515	1.2	0.00	49	0.04	0.7	5516
Djibouti	7100	32.3	0.01	88	0.2	15.1	438
Dominica	189	25.0	0.00	7	0.01	18.1	94
Dominican Republic	7820	16.1	0.02	724	1.2	10.8	22 143
Ecuador	77 248	27.2	0.14	2981	4.2	23.4	46 559
Egypt	4858	0.5	0.01	78	8.9	8.0	16 846
El Salvador	2515	12.0	0.01	89	0.9	13.5	12 658
Equatorial Guinea	9027	32.2	0.02	236	0.2	12.4	6309
Eritrea	25 126	20.7	0.05	242	1.2	32.3	3151
Estonia	378	0.8	0.00	60	0.003	0.2	21 241
Ethiopia	442 130	39.2	0.88	19 612	60.7	49.2	136 830
Faroe Islands, DK	182	13.3	0.00	133	0.02	30.6	182
Finland	31 909	9.5	0.19	1642	0.06	1.0	191 927
France	8995	1.6	0.03	478	0.4	0.6	350 208
French Guiana, FRA	48 790	53.6	0.09	1385	0.2	49.3	15 323
Gabon	98 244	36.7	0.19	3166	0.5	22.8	83 921
Gambia	3767	33.3	0.01	42	0.7	26.5	90
Georgia	8331	12.0	0.02	355	0.5	13.0	43 817
Germany	2225	0.6	0.01	76	0.3	0.3	271 768
Ghana	56 356	23.6	0.11	1002	6.7	20.0	17 778
Greece	3614	2.7	0.01	221	0.2	1.8	33 020
Greenland, DK	117 907	28.7	0.23	1634	0.001	2.1	19 113
Grenada	0	0.0	0.00	0	0	0.0	170
Guadeloupe, FRA	125	7.7	0.00	1	0.02	3.8	501
Guatemala	41 257	37.9	0.09	2564	5.7	31.8	48 699
Guinea	32 435	13.2	0.07	406	2.3	16.4	28 088
Guinea−Bissau	3090	8.6	0.01	36	5.5	26.2	290
Guyana	100 942	47.0	0.30	631	0.1	16.3	29 140
Haiti	7667	27.6	0.02	420	1.9	16.0	9167
Honduras	19 317	17.2	0.04	1212	1.3	12.3	43 029
Hong Kong, CN	48	4.6	0.00	3	0.005	0.1	477
Hungary	2942	3.2	0.01	68	0.3	2.8	13 651
Iceland	31 414	30.5	0.12	1519	0.03	7.1	14 523
India	310 956	9.5	0.65	7523	109.8	7.8	1754 623
Indonesia	911 635	47.5	1.76	36 809	94.5	34.3	288 948
Iran	24 740	1.5	0.06	509	2.8	3.2	366 828
Iraq	8445	1.9	0.02	55	2.2	5.0	74 681
Ireland	9592	13.6	0.04	277	1.0	19.1	33 416
Isle of Man, UK	114	20.0	0.00	0.4	0.01	16.0	0
Israel	147	0.7	0.00	7	0.03	0.4	1982
Italy	9336	3.1	0.03	407	1.01	1.7	183 129
Ivory Coast	31 476	9.8	0.06	424	4.2	14.9	54 656
Jamaica	5659	51.5	0.01	216	1.2	40.7	656
Japan	52 469	13.9	0.13	2173	11.1	9.0	41 706
Jordan	2126	2.4	0.01	23	0.7	5.8	2331
Kazakhstan	1 635 500	60.2	4.74	54 230	9.2	47.5	122 081
Kenya	255 340	43.8	0.50	7612	17.2	31.8	60 455
Korea DPR	51 455	42.7	0.13	2328	12.5	48.1	57 789
Korea	8458	8.6	0.02	341	5.1	9.8	70 814
Kyrgyzstan	59 449	39.0	0.15	1994	1.9	28.0	42 992
Laos	81 911	34.6	0.17	3247	2.4	31.5	94 854
Latvia	853	1.3	0.00	19	0.04	2.1	29 048
Lebanon	574	5.5	0.00	20	0.2	3.2	2152
Lesotho	3028	10.0	0.01	83	0.1	4.6	5761
Liberia	11 255	10.1	0.02	204	0.8	15.1	3061
Libya	414	0.02	0.00	17	0.01	0.2	4642
Liechtenstein	0	0.0	0.00	0	0	0.0	160
Lithuania	1673	2.6	0.01	55	0.06	2.1	15 831
Luxembourg	0	0.0	0.00	0	0	0.0	2519
Macao, CN	0	0.0	0.00	0	0	0.0	33
Macedonia	1104	4.4	0.00	43	0.1	5.2	4677
Madagascar	232 348	39.6	0.49	18 666	8.3	27.9	81 963
Malawi	86 415	72.9	0.15	5576	13. 7	67.2	7865
Malaysia	142 823	43.3	0.28	4111	12.3	36.4	80 026
Mali	129 739	10.5	0.27	113	6.7	29.8	4203
Malta	64	20.0	0.00	2	0.01	2.7	0
Martinique, FRA	103	9.1	0.00	6	0.02	4.1	103
Mauritania	66 330	6.4	0.14	34	1.1	23.6	239
Mauritius	243	13.0	0.00	6	0.2	12.8	243
Mexico	553 719	28.1	1.19	20 787	37.6	29.5	470 049
Moldova	3212	9.5	0.01	117	0.3	9.9	8148
Mongolia	195 640	12.5	0.56	4989	0.4	13.1	284 653
Montenegro	2677	19.9	0.007	118	0.14	22.2	5746
Morocco	80 605	18.1	0.17	1727	8.2	21.9	65 532
Mozambique	519 462	64.8	1.05	36 001	21.7	65.9	41 769
Myanmar	303 529	44.7	0.63	12 474	16.6	30.6	162 436
Namibia	124 317	15.1	0.27	2099	1.1	44.2	42 419
Nepal	34 158	24.3	0.08	1132	2.6	8.4	78 921
Netherlands	625	1.5	0.00	19	0.6	3.2	21 047
New Caledonia, FRA	10 474	55.0	0.02	1528	0.09	31.5	2232
New Zealand	154 257	57.4	0.40	16 762	1.7	32.7	59 223
Nicaragua	39 423	30.4	0.08	1084	1.6	23.2	38 794
Niger	203 968	16.1	0.40	29	14.2	54.0	612
Nigeria	326 370	35.3	0.64	4216	60.1	27.5	223 356
Norway	145 127	44.8	0.63	10 635	0.8	13.9	77 977
Oman	110	0.1	0.00	0.6	0.009	0.2	2206
Pakistan	45 927	5.7	0.11	1034	12.3	5.2	275 078
Panama	25 408	32.5	0.05	580	1.8	41.9	29 808
Papua New Guinea	323 115	69.8	0.62	16 267	5.4	53.6	36 431
Paraguay	107 035	26.3	0.23	3868	1.5	22.1	55 810
Peru	284 716	22.2	0.57	7451	3.0	8.9	436 963
Philippines	50 178	16.7	0.10	1532	14.8	12.9	89 520
Poland	1593	0.5	0.01	24	0.4	0.9	122 482
Portugal	1294	1.4	0.00	75	0.2	2.1	82 587
Puerto Rico, USA	3424	37.6	0.01	197	1.0	30.3	585
Reunion, FRA	502	20.0	0.00	42	0.07	6.9	419
Romania	10 530	4.4	0.03	326	0.5	2.6	58 367
Russia	6 438 070	37.7	26.3	371 321	25.3	17.5	4 979 045
Rwanda	15 601	59.2	0.03	793	8.8	63.8	1652
San Marino	0	0.0	0.00	0	0	0.0	60
Sao Tome and Principe	273	27.3	0.00	18	0.02	7.1	0
Saudi Arabia	16 653	0.9	0.04	98	0.9	2.5	12 450
Senegal	64 738	33.0	0.13	310	6.4	36.8	1153
Serbia	4336	5.0	0.012	116	0.54	6.0	7879
Sierra Leone	11 296	15.8	0.02	196	0.8	8.9	9357
Singapore	93	14.3	0.00	2	0.7	11.0	0
Slovakia	971	2.0	0.00	36	0.1	2.7	12 397
Slovenia	786	3.9	0.00	21	0.08	3.9	1511
Solomon Islands	17 949	63.1	0.04	1047	0.3	43.3	596
Somalia	175 394	27.5	0.35	3082	3.1	17.9	63 834
South Africa	110 901	9.1	0.25	3650	5.3	8.8	469 191
Spain	14 670	2.9	0.03	733	2.7	5.6	389 260
Sri Lanka	17 570	26.8	0.04	457	3.4	15.6	11 968
Sudan	331 122	13.2	0.68	1358	13.3	28.4	375 172
Suriname	109 300	66.9	0.19	2898	0.3	53.4	13 396
Svalbard, NOR	4295	6.9	0.01	33	0.0	3.5	5391
Swaziland	1523	8.8	0.00	111	0.1	8.4	11 956
Sweden	90 842	20.2	0.38	7013	0.2	1.8	267 055
Switzerland	4396	10.7	0.01	376	0.1	1.6	31 131
Syria	15 380	8.3	0.04	312	2.3	10.4	20 091
Taiwan, CN	4191	11.7	0.01	134	1.6	6.8	15 657
Tajikistan	23 411	16.4	0.05	610	2.2	22.1	26 484
Tanzania	370 036	39.2	0.70	22 929	27.7	42.2	309 865
Thailand	123 041	23.9	0.25	3886	14.4	20.1	240 437
Togo	8826	15.5	0.01	72	1.3	14.8	4829
Trinidad and Tobago	88	1.7	0.00	2	0.06	4.2	1061
Tunisia	18 112	11.1	0.04	278	2.0	16.4	20 882
Turkey	15 771	2.0	0.04	539	3.2	3.7	464 426
Turkmenistan	69 410	14.2	0.17	603	0.7	11.0	48 733
Turks and Caicos Islands, UK	195	45.5	0.00	42	0.0	0.78	39
Uganda	84 870	36.0	0.15	3262	24.9	52.7	31 562
Ukraine	194 215	32.2	0.57	9254	9.3	23.5	239 720
UK	31 004	12.7	0.01	952	2.4	3.7	57 579
USA	2 596 936	27.0	7.13	12 026	45.1	13.3	2 717 657
Uruguay	49 526	28.0	0.12	2022	0.4	11.3	50 030
Uzbekistan	143 847	32.2	0.35	1925	7.8	22.5	37 122
Vanuatu	4883	33.1	0.01	301	0.06	18.9	111
Venezuela	243 781	26.7	0.49	10 222	2.9	10.2	346 519
Vietnam	84 207	25.6	0.17	4513	12.7	12.9	198 822
Yemen	38 009	7.2	0.06	197	4.9	14.5	1638
Zambia	519 166	69.0	1.05	26 861	13.7	68.4	38 444
Zimbabwe	148 151	37.3	0.31	6939	6.9	42.1	48 567
Total	42 452 767	28.0	100	1 674 285	1207.5	15.1	38 948 417

Country	% territory	% global improving area	Total NPP gain ktC/41 years	LI affected people, millions	% total population in improving areas	Net NPP gain/loss ktC/41 years
Afghanistan	17.6	0.39	2861	10.3	25.0	1097
Albania	42.2	0.03	2055	0.9	31.6	1950
Algeria	3.0	0.17	2975	6.7	15.0	2092
Andorra	77.8	0.00	44	0.05	67.0	37
Angola	7.1	0.20	2717	0.8	2.3	−41 413
Antigua and Barbuda	50.0	0.00	30	0.06	53.5	30
Argentina	20.2	1.49	20 687	5.3	11.6	−12 824
Armenia	44.5	0.04	1290	0.8	27.1	1263
Australia	47.7	8.82	153 122	5.6	21.2	123 588
Austria	28.6	0.08	4626	2.1	23.7	4303
Azerbaijan	46.5	0.11	2726	3.4	33.2	2074
Bahamas	14.2	0.00	107	0.07	1.7	−132
Bangladesh	76.9	0.25	6198	122.8	71.8	6005
Barbados	0.0	0.00	4	0	0	−6
Belarus	38.0	0.29	7762	2.3	24.6	7357
Belgium	79.2	0.08	2303	7.4	63.5	2291
Belize	47.9	0.02	590	0.2	50.1	380
Benin	17.7	0.05	889	1.5	11.6	783
Bhutan	21.1	0.02	372	0.1	18.7	−142
Bolivia	38.6	0.94	12 703	4.9	40.2	3974
Bosnia and Herzegovina	36.2	0.06	1506	0.9	27.1	1124
Botswana	30.1	0.41	15 470	1.2	45.9	14 737
Brazil	28.8	5.41	160 665	53.9	25.0	60 007
Brunei	27.7	0.00	51	0.06	12.7	38
Bulgaria	9.3	0.03	1816	0.6	8.7	1731
Burkina Faso	2.4	0.01	88	0.3	1.3	−85
Burundi	21.1	0.01	237	2.1	16.2	−301
Cambodia	22.2	0.09	2192	4.4	26.2	−5359
Cameroon	47.8	0.48	13 601	9.3	33.2	11 856
Canada	19.6	7.90	127 273	4.5	11.7	−237 320
Cape Verde	22.2	0.00	2	0.1	25.2	−21
Central African Republic	37.7	0.51	8017	1.7	31.0	5924
Chad	1.7	0.05	77	0.7	4.1	−250
Chile	13.3	0.26	5513	1.2	6.2	−79
China	36.0	9.00	32 441	759.1	53.2	286 369
Colombia	43.1	1.06	24 141	24.3	46.8	15 259
Congo R	26.3	0.20	3236	0.9	15.4	−2703
Congo DR	15.7	0.79	9972	12.5	12.6	−59 030
Costa Rica	32.0	0.04	353	0.8	16.4	−618
Croatia	26.1	0.04	1864	0.6	14.5	1796
Cuba	48.0	0.12	4342	4.4	39.7	3927
Cyprus	45.5	0.01	527	0.2	17.5	501
Czech Republic	63.9	0.17	10 193	6.0	57.0	10 193
Denmark	12.8	0.02	932	1.1	18.4	884
Djibouti	2.0	0.00	0.4	0.1	0.7	−88
Dominica	12.5	0.00	9	0.01	14.4	2
Dominican Republic	45.4	0.05	1311	3.4	30.0	587
Ecuador	16.4	0.09	3517	3.2	17.7	537
Egypt	1.7	0.04	1728	15.9	14.3	1650
El Salvador	60.2	0.03	640	3.9	62.3	552
Equatorial Guinea	22.5	0.01	276	0.2	9.1	41
Eritrea	2.6	0.01	177	0.5	14.5	−66
Estonia	47.0	0.08	3239	0.2	14.2	3179
Ethiopia	12.1	0.30	7801	7.7	6.2	−11 811
Faroe Islands, DK	13.3	0.00	4	0.002	3.3	−9
Finland	57.0	0.90	35 403	2.3	42.1	33 761
France	64.0	1.10	42 840	33.7	52.1	42 362
French Guiana, FRA	16.8	0.03	400	0.05	17.4	−986
Gabon	31.4	0.18	4535	0.4	15.1	1369
Gambia	0.8	0.00	2	0.005	0.2	−40
Georgia	62.9	0.13	6844	1.5	40.2	6489
Germany	76.1	0.92	35 536	53.1	63.7	35 459
Ghana	7.5	0.04	556	0.8	2.5	−445
Greece	25.0	0.09	5648	1.8	17.5	5427
Greenland, DK	4.7	0.04	248	0	0.6	−1385
Grenada	50.0	0.00	19	0.05	42.5	19
Guadeloupe, FRA	30.8	0.00	46	0.09	21.7	45
Guatemala	44.7	0.11	3415	8.9	50.1	851
Guinea	11.4	0.06	1799	1.6	11.3	1393
Guinea−Bissau	0.8	0.00	11	0.02	1.1	−24
Guyana	13.6	0.06	785	0.04	5.4	−1846
Haiti	33.0	0.02	490	2.9	25.4	70
Honduras	38.4	0.10	3190	3.3	31.8	1979
Hong Kong, CN	45.5	0.00	76	3.0	40.2	72
Hungary	14.7	0.04	2052	1.4	13.7	1984
Iceland	14.1	0.06	656	0.02	6.0	−863
India	53.4	3.97	99 132	815.1	57.5	91 609
Indonesia	15.1	0.61	13 240	51.6	18.7	−23 569
Iran	22.3	0.92	14 297	29.9	33.7	13 789
Iraq	17.1	0.19	1905	14.0	31.5	1850
Ireland	47.6	0.14	3082	1.9	37.5	2805
Isle of Man, UK	0.0	0.00	0.4	0	0	−0.03
Israel	9.5	0.01	187	1.2	13.6	180
Italy	60.8	0.53	23 341	25.3	42.9	22 934
Ivory Coast	17.0	0.12	3499	3.4	12.0	3075
Jamaica	6.0	0.00	42	0.09	3.1	−174
Japan	11.0	0.11	5032	8.9	7.2	2860
Jordan	2.6	0.01	76	0.9	8.4	53
Kazakhstan	4.5	0.39	6527	0.6	3.0	−47 703
Kenya	10.4	0.13	2709	4.1	7.5	−4903
Korea DPR	48.0	0.16	2652	7.8	30.1	324
Korea	71.9	0.18	5236	21.9	42.2	4895
Kyrgyzstan	21.7	0.12	2079	0.8	11.4	84
Laos	40.1	0.21	3929	2.8	37.7	682
Latvia	45.0	0.11	3855	0.5	29.4	3832
Lebanon	20.7	0.01	171	0.3	6.0	151
Lesotho	19.0	0.01	308	0.8	34.6	225
Liberia	2.8	0.01	310	0.1	2.5	106
Libya	0.3	0.01	162	0.3	4.2	145
Liechtenstein	100.0	0.00	9	0.04	100	9
Lithuania	24.3	0.07	2113	0.7	26.1	2057
Luxembourg	97.8	0.01	194	0.6	90.9	194
Macao, CN	100.0	0.00	0.4	0.5	68.1	0.4
Macedonia	18.5	0.01	691	0.5	22.0	649
Madagascar	14.0	0.19	5036	3.3	11.2	−13 630
Malawi	6.64	0.01	361	0.8	4.1	−5214
Malaysia	24.3	0.17	2109	3.3	9.7	−2002
Mali	0.3	0.01	70	0.1	0.6	−43
Malta	0.0	0.00	4	0	0	2
Martinique, FRA	9.1	0.00	9	0.04	11.9	3
Mauritania	0.02	0.00	7	0.004	0.1	−28
Mauritius	13.0	0.00	57	0.06	4.3	52
Mexico	23.8	1.10	23 882	16.0	12.5	3095
Moldova	24.1	0.03	825	0.8	24.4	708
Mongolia	18.2	0.89	14 403	1.2	35.3	9415
Montenegro	42.7	0.016	342	0.194	31.0	224
Morocco	14.7	0.15	3162	5.1	13.6	1434
Mozambique	5.2	0.09	2831	1.0	3.0	−33 170
Myanmar	23.9	0.37	7792	11.8	21.7	−4682
Namibia	5.1	0.10	3206	0.04	1.4	1107
Nepal	56.1	0.11	4005	21.2	69.4	2874
Netherlands	50.7	0.06	2205	6.8	38.5	2186
New Caledonia, FRA	11.7	0.01	214	0.02	5.5	−1314
New Zealand	22.0	0.17	4361	0.3	5.1	−12 401
Nicaragua	30.0	0.08	1926	2.1	30.4	843
Niger	0.05	0.00	0.2	0.03	0.1	−28
Nigeria	24.2	0.48	8345	48.6	22.2	4128
Norway	24.1	0.37	6606	0.9	17.2	−4029
Oman	1.0	0.01	78	0.5	11.5	78
Pakistan	34.2	0.73	5542	104.8	44.5	4508
Panama	38.1	0.06	751	1.1	24.4	172
Papua New Guinea	7.9	0.08	1578	0.6	5.5	−14 689
Paraguay	13.7	0.13	1191	0.5	7.3	−2677
Peru	34.0	0.96	18 999	7.6	22.3	11 547
Philippines	29.8	0.19	4493	19.9	17.2	2961
Poland	39.2	0.43	20 079	12.8	32.2	20 055
Portugal	89.4	0.22	11 355	6.6	64.2	11 280
Puerto Rico, USA	6.4	0.00	50	0.3	7.9	−147
Reunion, FRA	16.7	0.00	37	0.07	7.0	−5
Romania	24.6	0.18	8444	7.4	37.6	8118
Russia	29.2	22.17	467 475	43.1	29.8	96 154
Rwanda	6.3	0.00	188	0.3	2.4	−605
San Marino	100.0	0.00	5	0.02	72.0	5
Sao Tome and Principe	0.0	0.00	2	0	0	−15
Saudi Arabia	0.6	0.03	252	0.5	1.3	155
Senegal	0.6	0.00	19	0.03	0.2	−291
Serbia	9.0	0.024	311	0.49	5.5	195
Sierra Leone	13.0	0.02	622	0.9	10.1	427
Singapore	0.0	0.00	1	0	0	−0.5
Slovakia	25.4	0.04	2924	1.4	24.8	2888
Slovenia	7.5	0.01	201	0.09	4.1	180
Solomon Islands	2.1	0.00	5	0.01	1.4	−1042
Somalia	10.0	0.14	1680	1.6	8.9	−1401
South Africa	38.5	1.16	33 315	21.1	35.3	29 665
Spain	77.1	1.09	43 661	24.2	50.9	42 929
Sri Lanka	18.2	0.03	641	1.9	8.6	184
Sudan	15.0	0.84	18 905	8.9	19	17 547
Suriname	8.2	0.03	283	0.04	6.9	−2615
Svalbard, NOR	8.7	0.01	69	0.0001	4.6	37
Swaziland	68.9	0.03	1390	1.0	82.1	1279
Sweden	59.4	1.21	39 679	3.5	33.5	32 665
Switzerland	75.4	0.10	4515	6.9	78.7	4139
Syria	10.9	0.05	1222	3.8	17.0	910
Taiwan, CN	43.5	0.04	1742	6.4	26.8	1608
Tajikistan	18.5	0.07	1116	3.1	30.7	506
Tanzania	32.8	0.64	22 027	10.2	15.6	−902
Thailand	46.8	0.54	15 666	24.3	33.9	11 779
Togo	8.5	0.01	237	0.5	5.9	164
Trinidad and Tobago	20.7	0.00	51	0.3	18.3	49
Tunisia	12.8	0.05	1091	3.1	24.8	813
Turkey	59.5	1.27	54 429	34.8	40.8	53 890
Turkmenistan	10.0	0.13	1111	0.6	9.7	508
Turks and Caicos Islands, UK	9.1	0.00	12	0.0001	0.1	−31
Uganda	13.4	0.06	2240	3.6	7.6	−1022
Ukraine	39.7	0.77	16 493	13.3	33.6	7239
UK	23.5	0.19	4288	8.5	12.6	3336
USA	28.2	8.14	128 583	43.8	13.0	8324
Uruguay	28.4	0.13	2698	0.3	8.5	676
Uzbekistan	8.3	0.01	938	4.5	13.0	−987
Vanuatu	0.8	0.00	2	0.003	0.9	−299
Venezuela	38.0	0.75	15 649	12.7	44.7	5427
Vietnam	60.3	0.44	12 832	61.3	62.4	8319
Yemen	0.3	0.00	47	0.3	0.8	−149
Zambia	5.1	0.08	1363	1.0	4.8	−25 498
Zimbabwe	12.4	0.11	4550	2.1	12.7	−2389
Total	26.2	100	2 368 151	2851	35.8	6936

* Countries or regions with no degradation are not listed

**Table 3:** Land degradation comparison between periods 1981–2003 and 1981–2021

Country	Degrading area 1981–2003 km^2^	Degrading area 1981–2021 km^2^	Change 1981–2003 to 1981–2021 km^2^	Change 1981–2003 to 1981–2021 %	NPP loss 1981–2003 ktC/23y	NPP loss 1981–2021 ktC/41y	Change 1981–2003 to 1981–2021 ktC	Change 1981–2003 to 1981–2021 ktC/y	Affected people 1981–2003 million	Affected people 1981–2021 million	Change 1981–2003 to 1981–2021 million
Afghanistan	7658	117 876	110 218	1439	63	1763	1700	40	0.7	8.8	8.1
Albania	2334	1565	− 769	− 33	47	105	58	0.5	0.1	0.03	− 0.1
Algeria	63 475	49 408	− 14 067	− 22	1977	883	− 1095	− 64	7.2	6.1	− 1.1
Andorra	281	52	− 229	− 82	3	7	5	0.1	0.02	0.01	− 0.01
Angola	828 029	835 156	7127	0.9	37 603	44 129	6527	− 559	9.3	14.0	4.8
Argentina	902 438	706 032	− 196 406	− 22	23 556	33 511	9955	− 206	14.5	9.5	− 5.0
Armenia	743	1442	699	94	14	27	13	0.1	0.08	0.1	0.04
Australia	1 994 268	1 302 627	− 691 641	− 35	46 905	29 533	− 17 371	− 1319	2.2	1.0	− 1.2
Austria	28 291	5011	− 23 280	− 823	2	322	320	8	1.7	0.1	− 1.6
Azerbaijan	2633	16 082	13 449	511	123	652	528	11	0.2	1.7	1.5
Bahamas	4130	3020	− 1110	− 27	195	239	44	− 3	0.02	0.01	− 0.01
Bangladesh	68 422	7083	− 61 339	− 90	2851	193	− 2658	− 119	72.7	5.0	− 67.7
Barbados	0	72	72	100	0	10	10	0.2	0	0.01	0.01
Belarus	4053	12 025	7972	197	82	405	33	6	0.3	0.6	0.4
Belgium	5404	379	− 5025	− 93	70	12	− 58	− 3	1.4	0.1	− 1.3
Belize	3026	5372	2346	78	66	210	144	2	0.04	0.08	0.04
Benin	14 155	21 917	7762	55	373	107	− 266	− 14	0.9	2.5	1.6
Bhutan	27 011	12 636	− 14 375	− 53	1706	514	− 1192	− 62	1.3	0.3	− 1.1
Bolivia	60 339	236 303	175 964	292	1656	8729	7073	141	1.5	2.2	0.6
Bosnia and Herzegovina	7737	8379	642	8	158	381	223	2	0.7	0.7	− 0.001
Botswana	97 831	45 353	− 52 478	− 54	4112	733	− 3378	− 161	0.5	0.1	− 0.3
Brazil	1 881 702	2 453 800	572 098	30	63 346	100 658	37 312	299	46.6	31.8	− 14.8
Brunei	2663	355	− 2308	− 87	128	13	− 115	− 5	0.3	0.02	− 0.3
Bulgaria	9139	3104	− 6035	− 66	178	85	− 93	− 6	0.9	0.2	− 0.7
Burkina Faso	9255	121 213	111 958	1209	124	174	50	− 1	1.1	9.5	8.4
Burundi	13 516	14 010	494	4	973	539	− 434	− 29	3.9	6.1	2.2
Cambodia	77 958	119 632	41 674	54	2525	7551	5026	74	3.6	9.0	5.4
Cameroon	151 605	78 044	− 73 561	− 49	9657	1745	− 7911	− 377	4.3	5.7	1.4
Canada	1 985 085	4 692 151	2 707 066	136	93 964	364 594	270 630	4807	5.5	7.8	2.3
Cape Verde	375	1344	969	258	12	24	12	0.1	0.1	0.1	0.01
Central African Republic	126 927	68 612	− 58 315	− 46	3702	2093	− 1609	− 110	0.9	0.90	0.02
Chad	52 735	140 009	87 274	166	627	328	− 299	− 19	1.0	6.1	5.0
Chile	77 230	115 417	38 187	49	1951	5592	3641	52	1.6	0.9	− 0.7
China	2 193 697	1 639 469	− 554 228	− 25	58 840	38 041	− 20 799	− 1630	457.2	118.9	− 338.3
Colombia	291 295	317 253	25 958	9	178	8883	− 9117	− 566	16.3	7.3	− 9.0
Comoros	181	0	− 181	− 100	18	0	− 18	− 1	0.1	0	− 0.1
Congo R	201 614	199 125	− 2489	− 1	20 091	594	− 14 151	− 723	1.9	2.4	0.5
Congo DR	1 346 914	1 641 579	294 665	22	3404	69 002	65 597	1535	32.1	56.8	24.7
Costa Rica	14 691	27 926	13 235	90	529	972	442	1	0.6	2.7	2.1
Croatia	2822	2142	− 680	− 24	28	68	40	0.4	0.3	0.1	− 0.2
Cuba	32 430	11 863	− 20 567	− 63	755	415	− 340	− 23	3.1	0.9	− 2.1
Cyprus	266	612	346	130	9	27	18	0.3	0.005	0.2	0.2
Czech Republic	11 218	111	− 11 107	− 99	304	1	− 303	− 13	1.4	0.1	− 1.3
Denmark	91	515	424	465	0.3	49	48	1	0.01	0.04	0.03
Djibouti	6107	7100	993	16	19	88	69	1	0.7	0.2	− 0.6
Dominica	126	189	63	50	9	7	− 2	− 0.2	0.004	0.01	0.01
Dominican Republic	18 507	7820	− 10 687	− 58	561	724	163	− 7	3.8	1.2	− 2.6
Ecuador	40 136	77 248	37 112	93	2401	2980	579	− 32	2.2	4.2	2.0
Egypt	36 514	4858	− 31 656	− 87	17	78	61	1	10.1	8.9	− 1.2
El Salvador	5585	2515	− 3070	− 55	235	89	− 146	− 8	1.1	0.9	− 0.3
Equatorial Guinea	15 376	9027	− 6349	− 41	1435	236	− 1198	− 57	0.2	0.2	− 0.04
Eritrea	15 573	25 126	9553	61	33	242	209	4	0.2	1.2	1.0
Estonia	423	378	− 45	− 10	4	60	56	1	0.009	0.003	− 0.006
Ethiopia	296 812	442 130	145 318	49	14 276	19 612	5336	− 142	20.7	60.7	40.0
Faroe Islands, DK	0	182	182	100	0	13	13	0.3	0	0.02	0.02
Falkland Islands	1635	0	− 1635	− 100	51	0	− 51	− 2	0.0004	0	− 0.0004
Finland	27 779	31 909	4130	15	328	1642	1314	26	0.2	0.06	− 0.1
France	46 691	8995	− 37 696	− 81	605	478	− 127	− 15	6.2	0.4	− 5.8
French Guiana, FRA	24 947	48 790	23 843	96	1033	1385	352	− 11	0.03	0.2	0.1
Gabon	172 865	98 244	− 74 621	− 43	23	3166	3143	76	0.5	0.5	0.07
Gambia	1396	3767	2371	169	26	42	16	− 0.1	0.03	0.07	0.07
Georgia	5647	8331	2684	48	141	355	214	3	0.6	0.5	− 0.1
Germany	32 479	2225	− 30 254	− 93	730	76	− 654	− 30	5.7	0.3	− 5.4
Ghana	50 365	56 356	5991	12	2521	1002	− 1519	− 85	4.5	6.7	2.2
Greece	6914	3614	− 3300	− 48	117	221	104	0.3	0.7	0.2	− 0.5
Greenland, DK	0	117 907	117 907	100	0	1634	1634	40	0	0.001	0.001
Guadeloupe, FRA	0	125	125	100	0	1	1	0.03	0	0.01	0.01
Guatemala	55 884	41 257	− 14 627	− 26	2867	2564	− 303	− 62	3.9	5.7	1.7
Guinea	91 415	32 435	− 58 980	− 65	2008	406	− 1602	− 8	4.1	2.2	− 1.8
Guinea − Bissau	18 851	3090	− 15 761	− 84	452	36	− 416	− 19	0.5	0.6	0.02
Guyana	93 448	100 942	7494	8	230	2631	2401	54	0.2	0.1	− 0.07
Haiti	11 821	7667	− 4154	− 35	383	420	37	− 6	2.8	1.9	− 1.0
Honduras	30 145	19 317	− 10 828	− 36	1451	1212	− 239	− 34	1.7	1.3	− 0.4
Hong Kong CN	0	48	48	100	0	3	3	0.07	0	5.1	5.1
Hungary	31 398	2942	− 28 456	− 91	766	68	− 697	− 32	2.8	0.3	− 2.5
Iceland	34 483	31 414	− 3069	− 9	2693	1519	− 1174	− 80	0.06	0.03	− 0.03
India	592 498	310 956	− 281 542	− 48	22 484	7523	− 14 960	− 794	177.4	109.8	− 67.7
Indonesia	1 028 942	911 635	− 117 307	− 11	67 680	36 810	− 30 870	− 2045	86.7	94.5	7.8
Iran	29 190	24 740	− 4450	− 15	282	509	226	0.1	2.6	2.8	0.2
Iraq	28 000	8445	− 19 555	− 70	1030	55	− 975	− 43	1.7	2.2	0.5
Ireland	6416	9592	3176	50	1363	277	− 1086	− 53	0.7	0.9	0.3
Isle of Man, UK	0	114	114	100	0	0.4	0.4	0.01	0	0.01	0.01
Israel	3085	147	− 2938	− 95	50	7	− 43	− 2	2.0	0.03	− 2.0
Italy	28 693	9336	− 19 357	− 68	696	407	− 289	− 20	4.3	1.0	− 3.3
Ivory Coast	117 595	31 476	− 86 119	− 73	6221	424	− 5797	− 260	6.3	4.2	− 2.1
Jamaica	3372	5659	2287	68	107	216	108	0.6	0.7	1.1	0.4
Japan	130 563	52 469	− 78 094	− 60	427	2173	− 2096	− 133	29.7	11.1	− 18.6
Jordan	13 574	2126	− 11 448	− 84	101	23	− 78	− 4	1.6	0.7	− 0.9
Kazakhstan	487 083	1 635 500	1 148 417	236	5308	54 230	48 922	1092	2.1	9.2	7.1
Kenya	104 994	255 340	150 346	143	6613	7612	999	− 102	11.8	17.2	5.4
Korea DPR	60 959	51 455	− 9504	− 16	2206	2328	122	− 39	10.1	12.5	2.4
Korea	54 091	8458	− 45 633	− 84	1571	340	− 1231	− 60	14.4	5.1	− 9.3
Kyrgyzstan	23 189	59 449	36 260	156	282	1994	1712	36	0.7	1.9	1.2
Laos	133 395	81 911	− 51 484	− 39	7233	3247	− 3986	− 235	3.3	2.4	− 0.9
Latvia	4416	853	− 3563	− 81	136	19	− 117	− 5	0.2	0.04	− 0.2
Lebanon	704	574	− 130	− 19	2	19	17	0.4	0.1	0.2	0.05
Lesotho	10 344	3028	− 7316	− 71	485	83	− 402	− 19	0.9	0.1	− 0.8
Liberia	50 500	11 255	− 39 245	− 78	2098	204	− 1894	− 86	1.4	0.8	− 0.6
Libya	12 672	414	− 12 258	− 97	86	17	− 69	− 3	0.4	0.01	− 0.4
Lithuania	2664	1673	− 991	− 37	55	55	10	− 1	0.1	0.06	− 0.07
Macedonia	1757	1104	− 653	− 37	33	43	10	− 0.4	0.03	0.1	0.08
Madagascar	163 843	232 348	68 505	42	6678	18 666	11 987	165	3.9	8.3	4.4
Malawi	30 869	86 415	55 546	179	1371	5576	4205	76	2.5	13.7	11.2
Malaysia	175 817	142 823	− 32 994	− 19	9258	4111	− 5147	− 302	10.4	12.3	1.9
Mali	35 637	129 739	94 102	264	358	113	− 245	− 13	0.9	6.7	5.9
Malta	0	64	64	100	0	2	21	0.05	0	0.01	0.01
Martinique, FRA	0	103	103	100	0	6	6	0.1	0	0.02	0.02
Mauritania	6301	66 330	60 029	953	18	34	16	0.06	0.07	1.1	1.1
Mauritius	0	243	243	100	0	6	6	0.1	0	0.2	0.2
Mexico	487 804	553 719	65 915	14	23 871	20 787	− 3084	− 531	36.2	37.6	1.4
Moldova	1751	3212	1462	84	32	117	85	1	0.1	0.3	0.2
Mongolia	66 559	195 640	129 081	194	624	4989	4365	95	0.07	0.4	0.4
Montenegro	4513	2677	− 1836	− 40.7	673	118	51	− 52	0.2	0.14	− 0.08
Morocco	67 399	80 605	13 206	19	2808	1727	− 1081	− 80	11.3	8.2	− 3.1
Mozambique	226 567	519 462	292 895	129	8398	36 001	27 603	513	5.2	21.7	16.6
Myanmar	358 887	303 529	− 55 358	− 15	23 625	12 474	− 11 151	− 723	23.6	16.6	− 7.1
Namibia	288 945	124 317	− 164 628	− 57	6388	2099	− 4289	− 225	0.7	1.1	0.5
Nepal	54 704	34 158	− 20 546	− 38	2375	1132	− 1243	− 76	13.3	2.6	− 10.8
Netherlands	7051	625	− 6426	− 91	92	19	− 73	− 4	2.8	0.6	− 2.2
New Caledonia, FRA	6902	10 474	3572	52	1008	1528	520	− 7	0.05	0.09	0.04
New Zealand	147 014	154 257	7243	5	6993	16 762	9769	105	1.0	1.7	0.7
Nicaragua	47 223	39 423	− 7800	− 17	2060	1084	− 976	− 63	1.7	1.6	− 0.1
Niger	22 563	203 968	181 405	804	142	29	− 113	− 5	0.8	14.2	13.3
Nigeria	91 443	326 370	234 927	257	3067	4216	1149	− 31	17.0	60.1	43.0
Norway	57 109	145 127	88 018	154	1213	10 635	9422	207	0.4	0.8	0.4
Oman	419	110	− 309	− 74	3	0.06	− 3	− 0.1	0.002	0.009	0.007
Pakistan	20 644	45 927	25 283	123	236	1034	798	15	5.8	12.3	6.5
Panama	8735	25 408	16 673	191	514	580	66	− 8	0.2	1.8	1.6
Papua New Guinea	205 500	323 115	117 615	57	16 275	16 267	− 8	− 311	2.0	5.4	3.4
Paraguay	66 704	107 035	40 331	61	1659	3868	2209	22	4.1	1.5	− 2.6
Peru	197 211	284 716	87 505	44	11 415	7451	− 3964	− 315	3.0	3.0	0.03
Philippines	132 275	50 178	− 82 097	− 62	4100	1532	− 2568	− 141	33.1	14.8	− 18.2
Poland	41 514	1593	− 39 921	− 96	891	24	− 867	− 38	5.5	0.4	− 5.1
Portugal	11 536	1294	− 10 242	− 89	233	75	− 159	− 8	0.4	0.2	− 0.2
Puerto Rico, USA	436	3424	2988	684	19	197	178	4	0.1	1.0	0.9
Reunion, FRA	175	502	327	186	6	42	358	1	0.04	0.07	0.03
Romania	16 902	10 530	− 6372	− 38	364	326	− 38	− 8	1.0	0.5	− 0.5
Russia	2 802 060	6 438 070	3 636 010	130	56 663	371 321	314 658	6593	8.6	25.3	16.7
Rwanda	11 404	15 601	4197	37	1053	793	− 260	− 26	3.3	8.8	5.5
Sao Tome and Principe	125	273	148	118	30	17	− 13	− 1	0.03	0.02	− 0.01
Saudi Arabia	8327	16 653	8326	100	4	98	93	2	0.5	0.9	0.4
Senegal	34 655	64 738	30 083	87	409	310	− 988	− 10	2.1	6.4	4.3
Serbia	5438	4336	− 1102	− 20.3	105	116	11	− 1743	0.46	0.54	0.07
Sierra Leone	35 902	11 296	− 24 606	− 69	1508	196	− 1312	− 61	2.1	0.8	− 1.3
Singapore	243	93	− 150	− 62	6	2	− 4	− 0.2	2.0	0.7	− 1.31
Slovakia	5066	971	− 4095	− 81	111	36	− 75	− 4	0.4	0.1	− 0.2
Slovenia	2492	786	− 1706	− 69	38	21	− 17	− 1	0.4	0.08	− 0.3
Solomon Islands	9065	17 949	8884	98	629	1047	419	− 2	0.2	0.3	0.1
Somalia	52 520	175 394	122 874	234	1834	3082	1248	− 5	1.5	3.1	1.6
South Africa	426 615	110 901	− 315 714	− 74	23 123	3650	− 19 473	− 916	20.5	5.3	− 15.3
Spain	63 266	14 670	− 48 596	− 77	1713	733	− 980	− 57	2.4	2.7	0.3
Sri Lanka	21 057	17 570	− 3487	− 17	635	457	− 178	− 16	4.8	3.4	− 1.4
Sudan	166 031	331 122	165 091	99	3628	1358	− 2270	− 125	3.3	13.3	10.0
Suriname	50 503	109 300	58 797	116	2102	2898	795	− 21	0.04	0.3	0.3
Svalbard, NOR	0	4295	4295	100	0	33	33	1	0	0.00	0.00
Swaziland	16 533	1523	− 15 010	− 91	1227	111	− 1116	− 51	0.9	0.1	− 0.9
Sweden	78 964	90 842	11 878	15	1594	7013	5419	102	0.8	0.2	− 0.6
Switzerland	4982	4396	− 586	− 12	106	376	269	5	0.5	0.1	− 0.2
Syria	11 327	15 380	4053	36	224	312	88	− 2	1.2	2.3	1.0
Taiwan, CN	0	4191	4191		0	134	134	3	0	1.6	1.6
Tajikistan	8412	23 411	14 999	178	104	610	506	10	0.2	2.2	2.0
Tanzania	386 256	370 036	− 16 220	− 4	22 604	22 929	325	− 424	15.3	27.7	12.4
Thailand	309 245	123 041	− 186 204	− 60	15 991	3886	− 12 104	− 600	37.0	14.4	− 22.6
Togo	11 064	8826	− 2238	− 20	299	72	− 227	− 11	0.7	1.3	0.7
Trinidad and Tobago	675	88	− 587	− 87	113	1	− 112	− 5	0.07	0.06	− 0.01
Tunisia	12 476	18 112	5636	45	398	278	− 120	− 11	1.5	2.0	0.5
Turkey	30 851	15 771	− 15 080	− 49	453	539	86	− 7	3.6	3.2	− 0.4
Turkmenistan	1273	69 410	68 137	5351	8	603	595	14	0.02	0.7	0.7
Turks and Caicos Islands, UK	92	195	103	111	16	42	26	0.3	0.00	0.00	0.00
Uganda	41 506	84 870	43 364	105	1513	3261	1748	14	4.1	24.9	20.8
Ukraine	47 414	194 215	146 801	310	1048	9254	8206	180	2.5	9.3	6.9
UK	23 506	31 004	7498	32	262	952	690	12	3.3	2.5	− 0.9
USA	1 983 886	2 596 936	613 050	31	39 673	120 259	80 586	1208	31.1	45.1	14.0
Uruguay	87 566	49 526	− 38 040	− 43	1875	2022	147	− 32	1.1	0.4	− 0.7
Uzbekistan	5974	143 847	137 873	2308	124	1925	1801	42	0.6	7.8	7.2
Vanuatu	2210	4883	2673	121	5	301	296	7	0.02	0.06	0.04
Venezuela	207 916	243 781	35 865	17	520	10 222	9702	227	2.2	2.9	0.7
Vietnam	134 026	84 207	− 49 819	− 37	343	4513	4170	95	28.1	12.7	− 15.4
Yemen	14 422	38 009	23 587	164	8	196	189	4	0.5	4.9	4.4
Zambia	454 630	519 166	64 536	14	19 900	26 861	6960	− 210	5.8	13.7	7.9
Zimbabwe	180 125	148 151	− 31 974	− 18	8862	6939	− 1923	− 216	5.4	6.9	1.4
Total	35 058 104	42 452 767	7 394 663	21	955 221	1 674 285	719 064	− 695	1537.7	1207.5	− 330.2

**Table 4:** Land improvement comparison between 1981–2003 and 1981–2021*

Country/ region	Improving area 1981–2003 km^2^	Improving area 1981–2021 km^2^	Change 1981–2003 to 1981–2021 km^2^	Change 1981–2003 to 1981–2021 %	NPP gain 1981–2003 ktC/23y	NPP gain 1981–2021 ktC/41y	Change 1981–2003 to 1981–2021	Change 1981–2003 to 1981–2021 ktC/y ktC	Affected people 1981–2003 million	Affected people 1981–2021 million	Change 1981–2003 to 1981–2021 million
Afghanistan	214 584	115 033	− 99 551	− 46	52.6	2860.7	2809.1	67.5	13.3	10.3	− 3.0
Albania	2815	12 125	9310	330	1.7	2055.2	2053.5	50.1	0.6	0.9	0.3
Algeria	60 359	72 409	12 050	20	14.9	2974.7	2959.8	71.9	3.9	6.8	2.9
Andorra	0	364	364	100	0	44.4	44.4	1.1	0	0.05	0.05
Angola	40 653	88 384	47 731	117	19.3	2717.3	2698.0	65.4	1.2	0.8	− 0.4
Antigua and Barbuda	0	220	220	100	0	29.6	29.6	0.7	0	0.05	0.05
Argentina	499 109	558 455	59 346	12	237.1	20 687.3	20 450.3	494.3	2.6	5.3	2.7
Armenia	1906	13 252	11 346	595	1.3	1290.5	1289.2	31.4	0.2	0.8	0.6
Australia	1 627 934	3 667 202	2 039 268	125	911.0	153 121.7	152 210.7	3695.0	3.0	5.6	2.6
Austria	1807	23 959	22 152	1226	134.6	4625.8	4624.4	112.8	0.2	2.1	2.0
Azerbaijan	848	40 306	39 458	4656	0.4	2725.6	2725.2	66.5	0.2	3.4	3.3
Bahamas	0	1975	1975	100	0	107.5	107.5	2.6	0	0.007	0.007
Bahrain	311	0	− 311	− 100	0	0	0	0	0.03	0	− 0.03
Bangladesh	6487	110 667	104 180	1606	2.7	6197.7	6195.0	151.0	5.3	122.8	117.6
Belarus	122 831	78 854	− 43 977	− 36	93.5	7762.3	7668.8	185.3	4.7	2.3	− 2.3
Belgium	0	24 170	24 170	100	0	2302.8	2302.8	56.2	0	7.4	7.4
Belize	2316	11 003	8687	375	1.0	590.3	589.3	14.4	0.02	0.2	0.2
Benin	50 213	19 947	− 30 266	− 60	45.9	889.2	843.4	19.7	2.8	1.5	− 1.2
Bhutan	355	9909	9554	2692	0.9	372.0	371.1	9.0	0.03	0.1	0.1
Bolivia	244 382	424 512	180 130	74	78.9	12 703.4	12 624.6	306.4	2.4	4.9	2.5
Bosnia and Herzegovina	47	18 531	18 484	39 127	0	1505.8	1505.8	36.7	0.006	0.9	0.9
Botswana	376 664	180 506	− 196 158	− 52	176.4	15 470.3	15 293.9	369.7	0.7	1.2	0.5
Brazil	1 659 571	2 451 035	791 464	48	148.0	160 665.2	159 185.2	3854.3	21.3	53.9	32.6
Brunei	0	1598	1598	100	0	51.1	51.1	1.2	0	0.06	0.06
Bulgaria	37 092	10 261	− 26 831	− 72	20.4	1815.8	1795.4	43.4	2.3	0.6	− 1.7
Burkina Faso	97 636	6473	− 91 163	− 93	73.0	88.2	15.2	− 1.0	3.9	0.3	− 3.6
Burundi	2007	5869	3862	192	1.1	237.3	236.1	5.7	0.6	2.1	1.5
Cambodia	4269	40 212	35 943	842	2.1	2192.3	2190.2	53.4	0.2	4.4	4.2
Cameroon	72 839	227 221	154 382	212	72.4	13 601.4	13 529.0	328.6	2.3	9.3	7.0
Canada	671 781	1 950 420	1 278 639	190	300.2	127 273.4	126 973.2	3091.2	1.4	4.5	3.1
Cape Verde	0	896	896	100	0	2.5	2.5	0.06	0	0.1	0.1
Central African Republic	73 564	234 838	161 274	219	39.8	8017.3	7977.5	193.8	0.4	1.7	1.3
Chad	72 762	21 335	− 51 427	− 71	37.3	77.2	39.9	0.3	1.0	0.7	− 0.3
Chile	177 982	100 782	− 77 200	− 43	246.0	5513.0	5266.9	− 123.8	1.4	1.2	− 0.2
China	807 802	3 449 516	2 641 714	327	316.7	324 410.6	324 093.8	7898.7	94.9	759.1	664.2
Colombia	241 729	491 232	249 503	103	262.8	24 141.4	23 878.6	577.4	4.4	24.3	19.9
Comoros	98	0	− 98	− 100	0.1	0	− 0.1	− 0.004	− .03	0	− 0.03
Congo DR	195 400	368 562	173 162	89	148.9	9971.7	9822.8	236.7	5.3	12.5	7.2
Congo R	41 940	90 018	48 078	115	32.1	3235.9	3203.7	77.5	0.3	0.9	0.6
Costa Rica	5526	16 172	10 646	193	724.1	353.4	346.1	8.3	0.3	0.8	0.5
Croatia	1098	14 735	13 637	1242	0.8	1864.2	1863.4	45.4	0.1	0.6	0.5
Cuba	1417	53 180	51 763	3653	0.4	4341.7	4341.3	105.9	0.08	4.4	4.4
Cyprus	345	4205	3860	1120	0.09	527.2	527.2	12.9	0.02	0.2	0.2
Czech Republic	6175	50 385	44 210	716	4.4	10 194.0	10 189.1	248.4	0.8	6.0	5.1
Denmark	356	5516	5160	1451	0.2	932.4	932.1	22.7	0.02	1.1	1.1
Djibouti	6581	438	− 6143	− 93	1.5	0.4	− 1.1	− 0.06	0.05	0.007	− 0.04
Dominica	68	94	26	38	0.1	8.6	8.5	0.2	0.004	0.01	0.01
Dominican Republic	1529	22 143	20 614	1348	0.7	1310.8	1310.0	31.9	0.2	3.4	3.1
Ecuador	28 133	46 559	18 426	66	58.8	3517.5	3458.7	83.2	2.1	3.2	1.1
Egypt	3158	16 846	13 688	433	1.5	1728.2	1726.8	42.1	1.4	15.9	14.5
El Salvador	1870	12 658	10 788	577	1.0	640.2	639.2	15.6	0.3	3.9	3.7
Equatorial Guinea	3377	6309	2932	87	3.5	276.3	272.8	6.6	0.06	0.2	0.09
Eritrea	9792	3151	− 6641	− 68	7.7	176.7	169.0	4.0	0.9	0.5	− 0.4
Estonia	5239	21 241	16 002	305	2.7	3238.7	3236.0	78.9	0.09	0.2	0.1
Ethiopia	306 748	136 830	− 169 918	− 55	166.3	7800.6	7634.3	183.0	7.1	7.7	0.6
Faroe Islands, DK	0	182	182	100	0	4.0	4.0	0.1	0	0.002	0.002
Finland	433	191 927	191 494	44 250	0.09	35 402.6	35 402.5	863.5	0.02	2.3	2.3
France	7296	350 208	342 912	4700	4.3	42 839.9	42 835.5	1044.7	0.5	33.7	33.2
French Guiana	4140	15 323	11 183	270	4.2	399.8	395.6	9.6	0.01	0.05	0.04
Gabon	32 999	83 921	50 922	154	27.4	4534.7	4507.3	109.4	0.5	0.4	− 0.2
Gambia	4454	90	− 4364	− 98	1.7	1.7	0.005	− 0.03	0.6	0.05	− 0.5
Georgia	5076	43 817	38 741	763	4.3	6843.6	6839.3	166.7	0.7	1.5	0.8
Germany	4182	271 768	267 586	6398	2.3	35 535.8	35 533.5	866.6	0.6	53.1	52.5
Ghana	101 775	17 778	− 83 997	− 83	79.0	556.3	477.3	10.1	6.8	0.8	− 5.9
Greece	8188	33 020	24 832	303	3.2	5647.9	5644.8	137.6	0.7	1.8	1.1
Greenland DK	0	19 113	19 113	100	0	248.5	248.5	6.1	0	0.0003	0.0003
Grenada	0	170	170	100	0	19.4	19.4	0.4	0	0.05	0.05
Guadeloupe FRA	0	501	501	100	0	45.7	45.7	1.1	0	0.09	0.09
Guatemala	2098	48 699	46 601	2222	1.0	3415.3	3414.3	83.3	0.07	8.9	8.9
Guinea	25 842	28 088	2246	9	11.9	1799.4	1787.5	43.4	0.7	1.6	0.8
Guinea−Bissau	4181	290	− 3891	− 93	1.3	11.5	10.2	0.2	0.1	0.02	− 0.09
Guyana	24 388	29 140	4752	20	41.0	785 4	744.6	17.4	0.04	0.04	0.003
Haiti	898	9167	8269	921	0.3	490.0	489.7	11.9	0.2	2.9	2.7
Honduras	9022	43 029	34 007	377	3.2	3190.3	3187.2	77.7	0.4	3.3	2.9
Hong Kong CN	0	477	477	100	0	75.6	75.6	1.8	0	3.0	3.0
Hungary	5519	13 651	8132	147	2.9	2052.3	2049.4	49.9	0.7	1.4	0.7
Iceland	1596	14 523	12 927	810	0.3	655.6	655.3	16.0	0.001	0.02	0.02
India	1 092 674	1 754 623	661 949	61	594.8	99 132.0	98 537.3	2392.0	279.4	815.1	535.7
Indonesia	182 690	288 948	106 258	58	95.3	13 240.5	13 145.2	318.8	18.0	51.6	33.6
Iran	186 294	366 828	180 534	97	44.5	14 297.3	14 252.7	346.8	9.3	29.9	20.5
Iraq	50 623	74 681	24 058	48	20.0	1904.9	1884.9	45.6	7.7	14.0	6.3
Ireland	40	33 416	33 376	83 018	0.02	3081.7	3081.7	75.2	0.002	1.9	1.9
Israel	61	1982	1921	3168	0.06	187.1	187.1	4.6	0.003	1.2	1.2
Italy	29 068	183 129	154 061	530	20.5	23 340.9	23 320.4	568.4	3.7	25.3	21.6
Ivory Coast	0	54 656	54 656	100	0	3498.9	3498.9	85.3	0	3.4	3.4
Jamaica	131	656	525	401	0.1	41.7	41.6	1.0	0.03	0.09	0.06
Jammu and Kashmir	4050	0	− 4050	− 100	6.3	0	− 6.3	− 0.3	1.8	0	− 1.8
Japan	1793	41 706	39 913	2226	1.1	5032.4	5031.26	122.7	0.2	8.9	8.7
Jordan	786	2331	1545	197	0.2	76.3	76.1	1.9	0.2	0.9	0.8
Kazakhstan	667 838	122 081	− 545 757	− 82	147.1	6527.4	6380.3	152.8	3.9	0.6	− 3.3
Kenya	317 890	60 455	− 257 435	− 81	313.8	2709.0	2395.3	52.4	5.1	4.1	− 1.1
Korea DPR	306	57 789	57 483	18 770	0.08	2652.2	2652.1	64.7	0.07	7.8	7.8
Korea	0	70 814	70 814	100	0	5235.9	5235.9	127.7	0	21.9	21.9
Kyrgyzstan	37 397	42 992	5595	15	17.4	2078.5	2061.1	49.9	0.5	0.8	0.2
Laos	4573	94 854	90 281	1974	4.1	3929.0	3924.9	95.7	0.08	2.8	2.8
Latvia	31 343	29 048	− 2295	− 7	19.2	3854.8	3835.6	93.3	0.6	0.5	− 0.07
Lebanon	2433	2152	− 281	− 12	1.4	170.5	169.1	4.1	0.3	0.3	0.06
Lesotho	6423	5761	− 662	− 10	1.8	307.9	306.1	7.4	0.6	0.8	0.2
Liberia	6724	3061	− 3663	− 55	7.5	310.2	302.72	7.2	0.4	0.1	− 0.3
Libya	8454	4642	− 3812	− 45	1.7	162.2	160.5	3.9	0.9	0.3	− 0.6
Liechtenstein	0	160	160	100	0	8.6	8.6	0.2	0	0.04	0.04
Lithuania	21 661	15 831	− 5830	− 27	14.6	2112.5	2097.9	50.9	0.8	0.7	− 0.04
Luxembourg	0	2519	2519	100	0	194.4	194.4	4.7	0	0.6	0.6
Macao, CN	0	33	33	100	0	0.4	4.1	0.01	0	0.5	0.5
Macedonia	2242	4677	2435	109	1.1	691.4	690.3	16.8	0.2	0.5	0.2
Madagascar	63 548	81 963	18 415	29	26.8	5036.1	5009.3	121.7	1.2	3.3	2.1
Malawi	13 698	7865	− 5833	− 43	4.2	361.4	357.2	8.6	1.7	0.8	− 0.9
Malaysia	15 933	80 026	64 093	402	13.7	2108.7	2095.0	50.8	0.6	3.3	2.7
Mali	205 629	4203	− 201 426	− 98	89.9	69.8	− 20.1	− 2.2	5.4	0.1	− 5.2
Martinique, FRA	0	103	103	100	0	8.6	8.6	0.2	0	0.04	0.04
Mauritania	2300	239	− 2061	− 90	0.4	6.5	6.1	0.1	0.04	0.004	− .04
Mauritius	321	243	− 78	− 24	0.2	57.5	57.3	1.4	0.2	0.06	− 0.1
Mexico	225 347	470 049	244 702	109	78.7	23 881.6	23 802.9	579.1	5.8	16.0	10.1
Moldova	20 350	8148	− 12 202	− 60	11.2	825.2	814.0	19.6	2.1	0.8	− 1.3
Mongolia	213 697	284 653	70 956	33	78.2	14 403.4	14 325.2	347.9	0.2	1.2	1.0
Montenegro	0	5746	5746	100	0	342	342	8.3	0	0.19	0.19
Morocco	94 552	65 532	− 29 020	− 31	34.6	3161.8	3127.1	75.6	6.8	5.1	− 1.7
Mozambique	153 790	41 769	− 112 021	− 73	54.9	2831.0	2776.0	66.7	4.1	1.0	− 3.1
Myanmar	24 292	162 436	138 144	569	19.8	7792.3	7772.5	189.2	1.1	11.8	10.7
Namibia	130 240	42 419	− 87 821	− 67	50.6	3205.9	3155.2	76.0	0.2	0.04	− 0.1
Nepal	4605	78 921	74 316	1613	3.2	4005.4	4002.2	97.6	0.8	21.2	20.4
Netherlands	0	21 047	21 047	100	0	2205.2	2205.2	53.8	0	6.8	6.8
New Caledonia FRA	810	2232	1422	176	0.9	214.1	213.2	5.2	0.005	0.02	0.01
New Zealand	7735	59 223	51 488	666	4.3	4360.8	4356.5	106.2	0.03	0.3	0.2
Nicaragua	14 305	38 794	24 489	171	6.5	1926.4	1919.8	46.7	0.5	2.1	1.6
Niger	101 911	612	− 101 299	− 99	23.4	0.2	− 23.2	− 1.0	1.8	0.03	− 1.8
Nigeria	160 792	223 356	62 564	39	150.5	8344. 5	8194.0	197.0	22.0	48.6	26.6
Norway	16 739	77 977	61 238	366	7.0	6606.0	6598.9	160.8	0.07	0.9	0.9
Oman	9922	2206	− 7716	− 77	1.4	78.3	76.9	1.8	0.4	0.5	0.1
Pakistan	291 709	275 078	− 16 631	− 6	90.2	5542.3	5452.0	131.3	68.5	104.8	36.3
Panama	20 843	29 808	8965	43	15.5	751.5	736.0	17.6	0.7	1.1	0.4
Papua New Guinea	64 377	36 431	− 27 946	− 43	28.2	1578.4	1550.1	37.3	0.8	0.6	− 0.3
Paraguay	91 068	55 810	− 35 258	− 39	40.8	1191.3	1150.5	27.3	0.4	0.5	0.08
Peru	272 800	436 963	164 163	60	405.4	18 998.5	18 593.1	445.8	3.7	7.6	3.9
Philippines	7410	89 520	82 110	1108	3.3	4493.0	4489.7	109.4	1.4	19.9	18.5
Poland	13 110	122 482	109 372	834	8.6	20 079.0	20 070.4	489.4	1.0	12.8	11.8
Portugal	265	82 587	82 322	31 009	0.2	11 354.6	11 354.4	276.9	0.1	6.6	6.5
Puerto Rico, USA	548	585	37	7	0.2	50.4	50.1	1.2	0.09	0.3	0.2
Qatar	243	0	− 243	− 100	0.004	0	− 0.004	− 0.15	0.004	0	− 0.004
Reunion, FRA	152	419	267	175	0.2	36.5	36.3	0.9	0.04	− .07	0.03
Romania	81 042	58 367	− 22 675	− 28	50.0	8444.1	8394.0	203.8	8.8	7.4	− 1.4
Russia	1 671 259	4 979 045	3 307 786	198	819.4	467 474.7	466 655.2	11 366.2	23.8	43.1	19.2
Rwanda	2011	1652	− 359	− 18	1.1	188.1	187.0	4.5	0.6	0.3	− 0.2
San Marino	0	60	60	100	0	5.1	5.1	0.1	0	0.02	0.02
Sao Tome and Principe	77	0	− 77	− 100	0.003	0	− 0.003	0	0.01	0	− 0.01
Saudi Arabia	17 833	12 450	− 5383	− 30	4.6	252.3	247.6	6.0	0.7	0.5	− 0.2
Senegal	72 830	1153	− 71 677	− 98	15.0	19.5	4.5	− 0.2	1.8	0.03	− 1.8
Serbia	21 445	7879	− 13 566	− 63.3	11.6	311.2	299.6	7.1	2.1	0.49	− 1.65
Sierra Leone	7370	9357	1987	27	3.1	622.2	619.1	15.0	0.4	0.9	0.4
Slovakia	1381	12 397	11 016	798	1.0	2924.0	2923.0	71.3	0.1	1.4	1.3
Slovenia	0	1511	1511	100	0	201.4	201.4	4.9	0	0.09	0.09
Solomon Islands	3600	596	− 3004	− 83	4.0	4.6	0.1	− .05	0.03	0.01	− 0.02
Somalia	229 512	63 834	− 165 678	− 72	138.0	1680.1	1542.5	35.0	3.6	1.6	− 2.0
South Africa	332 475	469 191	136 716	41	114.1	33 315.4	33 201.3	807.6	3.6	21.1	17.6
South Sudan	116 195	0	− 116 195	− 100	134.6	0	− 134.6	− 5.9	1.4	0	− 1.4
Spain	88 846	389 260	300 414	338	66.4	43 661.5	43 595.1	1062.0	3.9	24.2	20.3
Sri Lanka	1833	11 968	10 135	553	1.1	641.1	640.0	15.6	0.3	1.9	1.6
Sudan	129 811	375 172	245 361	189	73.7	18 904.6	18 830.8	457.9	2.1	8.9	6.8
Suriname	15 241	13 396	− 1845	− 12	166	282.6	266.0	6.2	0.007	0.04	0.04
Svalbard, NOR	0	5391	5391	100	0	69.3	69.3	1.7	0	0.0001	0.0001
Swaziland	0	11 956	11 956	100	0	1389.8	1389.8	33.9	0	1.0	1.0
Sweden	1705	267 055	265 350	15 560	0.8	39 678.7	39 677.8	967.7	0.008	3.5	3.5
Switzerland	1085	31 131	30 046	2768	1.4	4515.0	4513.6	110.1	0.03	6.9	6.8
Syria	52 713	20 091	− 32 622	− 62	23.8	1222.2	1198.4	28.8	7.0	3.8	− 3.2
Taiwan, CN	0	15 657	15 657	100	0	1741.8	1741.8	42.5	0	6.4	6.4
Tajikistan	40 349	26 484	− 13 865	− 34	12.3	1116.0	1103.7	26.7	2.6	3.1	0.4
Tanzania	118 767	309 865	191 098	161	59.9	22 027.3	21 967.4	534.6	4.1	10.2	6.1
Thailand	17 474	240 437	222 963	1276	8.1	15 665.8	15 657.7	381.7	2.29	24.3	22.1
East Timor	135	0	− 135	− 100	0.03	0	− 0.03	− 0.002	0.01	0	− 0.01
Togo	20 529	4829	− 15 700	− 77	15.1	236.6	221.5	5.1	1.9	0.5	− 1.3
Trinidad and Tobago	147	1061	914	624	0.1	50.5	50.7	1.2	0.02	0.37	0.3
Tunisia	14 377	20 882	6505	45	8.7	1091.2	1082.5	26.2	1.8	3.1	1.3
Turkey	217 418	464 426	247 008	114	142.4	54 428.8	54 286.3	1321.3	21.8	34.8	13.0
Turkmenistan	104 829	48 733	− 56 096	− 54	21.7	1110.9	1089.3	26.2	1.3	0.6	− 0.7
Turks and Caicos Islands, UK	0	39	39	100	0	11.8	11.8	0.3	0	0.00006	0.00006
UK	79	57 579	57 500	72 699	0.8	4288.0	4287.9	104.6	0.005	0.8	0.8
Uganda	21 010	31 562	10 552	50	10.7	2239.7	2229.0	54.2	2.1	3.6	1.4
Ukraine	178 281	239 720	61 439	35	96.0	16 492.9	16 396.9	398.1	12.6	13.3	0.8
United Arab Emirates	605	0	− 605	− 100	0.01	0	− 0.01	0	0.03	0	− 0.03
USA	1 641 111	2 717 657	1 076 546	66	563.9	128 583.3	128 019.4	3111.7	23.9	43.8	19.8
Uruguay	2588	50 030	47 442	1833	1.2	2698.0	2696.7	65.8	0.1	0.3	0.1
Uzbekistan	134 121	37 122	− 96 999	− 72	44.6	938.2	893.6	20.9	10.4	4.5	− 5.9
Vanuatu	256	111	− 145	− 57	0.2	2.1	1.9	0.04	0.002	0.003	0.0005
Venezuela	226 087	346 519	120 432	533	20.3	15 648.5	15 445.1	372.8	4.0	12.7	8.7
Vietnam	8967	198 822	189 855	2117	7.9	12 832.3	12 824.5	312.6	2.9	61.3	58.4
West Bank	50	0	− 50	− 100	0.02	0	− 0.02	− 1.0	0.005	0	− 0.005
Yemen	4247	1638	− 2609	− 61	98.8	47.1	− 51.7	− 3.1	0.6	0.3	− 0.4
Zambia	79 386	38 444	− 40 942	− 52	27.0	1362.8	1335.8	32.1	1.42	1.0	− 0.47
Zimbabwe	66 955	48 567	− 18 388	− 28	27.1	4549.7	4522.7	109.8	1.5	2.1	0.6
Total	18 725 478	38 948 417	20 222 939	108	16 648.0	2 368 151.5	2 351 503.8	57 036.0	831.0	2851.0	2019.9

* Countries or regions with no improvement are not listed

Breaking down the degrading land by cover type (ESA Copernicus 2023) reveals that 33% of all degraded land is scrub (shrub and herbaceous land in the ESA Copernicus listing), 24% is broadleaved forest, 21% is needle-leaved forest, 13% is grassland, and 9% is cropland (Table 5). As a proportion of each land cover type, degradation affects 30% of global scrublands, 32% of broadleaved forest, 38% of needle-leaved forest, 30% of grassland, and 24% of cropland. Needle-leaved forest stands out with the highest proportion of land affected because of the incidence of megafires in boreal forests: it might be overly optimistic to take the lower proportion of cropland affected as a policy success since severely degraded cropland often ceases to be classified as such because it is not cropland anymore.

**Table 5:** Global degrading and improving areas by land cover 1981–2021

Code	Land cover ESA Copernicus LC 2020	Total pixels, TP 5′ × 5′	Degrading pixels, DP 5′ × 5′	DP/TP %	DP/TDP* %	Improving pixels, IP 5′ × 5′	IP/TP %	IP/TIP** %
10	Cropland, rainfed	116 901	30 198	25.8	5.4	37 412	32.0	7.18
11	Herbaceous	113 294	29 489	26.0	5.3	37 848	33.4	7.26
12	Tree or shrub	2769	344	12.4	0.06	1307	47.2	0.25
20	Cropland, irrigated or post-flooding	32 985	4382	13.3	0.8	17 791	53.9	3.41
30	Mosaic cropland (> 50%) / natural vegetation (tree)	53 149	13 741	25.9	2.5	17 455	32.8	3.35
40	Mosaic natural vegetation (tree)	49 532	13 646	27.5	2.5	15 857	32.0	3.04
50	Tree cover, broadleaved, evergreen, closed to open (> 15%)	152 214	61 022	40.1	11.0	36 308	23.9	6.97
60	Tree cover, broadleaved, deciduous, closed to open (> 15%)	94 178	24 338	25.8	4.4	35 811	38.0	6.87
61	Tree cover, broadleaved, deciduous, closed (> 40%)	12 303	3413	27.7	0.6	4202	34.2	0.81
62	Tree cover, broadleaved, deciduous, open (15–40%)	43 624	14 336	32.9	2.6	11 995	27.5	2.30
70	Tree cover, needle-leaved, evergreen, closed to open (> 15%)	125 879	32 688	26.0	5.9	54 741	43.5	10.50
71	Tree cover, needle-leaved, evergreen, closed (> 40%)	50 591	30 318	59.9	5.5	10 216	20.2	1.96
72	Tree cover, needle-leaved, evergreen, open (15–40%)	21	9	42.9	0.002	8	38.1	0.002
80	Tree cover, needle-leaved, deciduous, closed to open (> 15%)	122 069	50 886	41.7	9.1	30 476	25.0	5.85
81	Tree cover, needle-leaved, deciduous, closed (> 40%)	50	42	84.0	0.01	2	4.0	0.00
90	Tree cover, mixed leaf type (broadleaved and needle-leaved)	41 711	7403	17.7	1.3	20 275	48.6	3.89
100	Mosaic tree and shrub (> 50%) / herbaceous (< 50%)	64 709	22 209	34.3	4.0	21 231	32.8	4.07
110	Mosaic herbaceous (> 50%) / tree and shrub (< 50%)	17 340	6735	38.8	1.2	4290	24.7	0.82
120	Shrubland	156 515	42 938	27.4	7.7	38 586	24.7	7.40
121	Shrubland evergreen	3389	1272	37.5	0.2	1182	34.9	0.23
122	Shrubland deciduous	36 550	13 343	36.5	2.4	10 322	28.2	1.98
130	Grassland	197 266	54 845	27.8	9.9	51 572	26.1	9.89
140	Lichens and mosses	47 172	18 106	38.4	3.3	7534	16.0	1.45
150	Sparse vegetation (tree/shrub & herbaceous cover) (< 15%)	173 288	56 342	32.5	10.1	35 602	20.5	6.83
152	Sparse shrub (< 15%)	1138	382	33.6	0.1	210	18.5	0.04
153	Sparse herbaceous (< 15%)	4027	963	23.9	0.2	739	18.4	0.14
160	Tree cover, flooded, fresh or brackish water	13 005	7082	54.5	1.3	2483	19.1	0.48
170	Tree cover, flooded, saline water	2714	561	20.7	0.1	389	14.3	0.07
180	Shrub or herbaceous cover	38 427	13 329	34.7	2.4	12 184	31.7	2.34
190	Urban areas	12 241	2325	19.0	0.4	3205	26.2	0.61
Total		1 779 051	556 687	31.3	100	521 233	29.3	100

* TDP – Total degrading pixels

** TIP – Total improving pixels

Of the areas degrading over 1981–2003, 41% have continued to degrade through 1981–2021 (Fig. S1): mainly in the Democratic Republic of Congo (DR Congo), Angola, Zambia, swaths of boreal forest in Siberia and North America, mainland Southeast Asia, the East Indies, parts of North-central Australia, the Eurasian Steppes, and the Pampas and Chaco in South America.

**Fig. 4:**
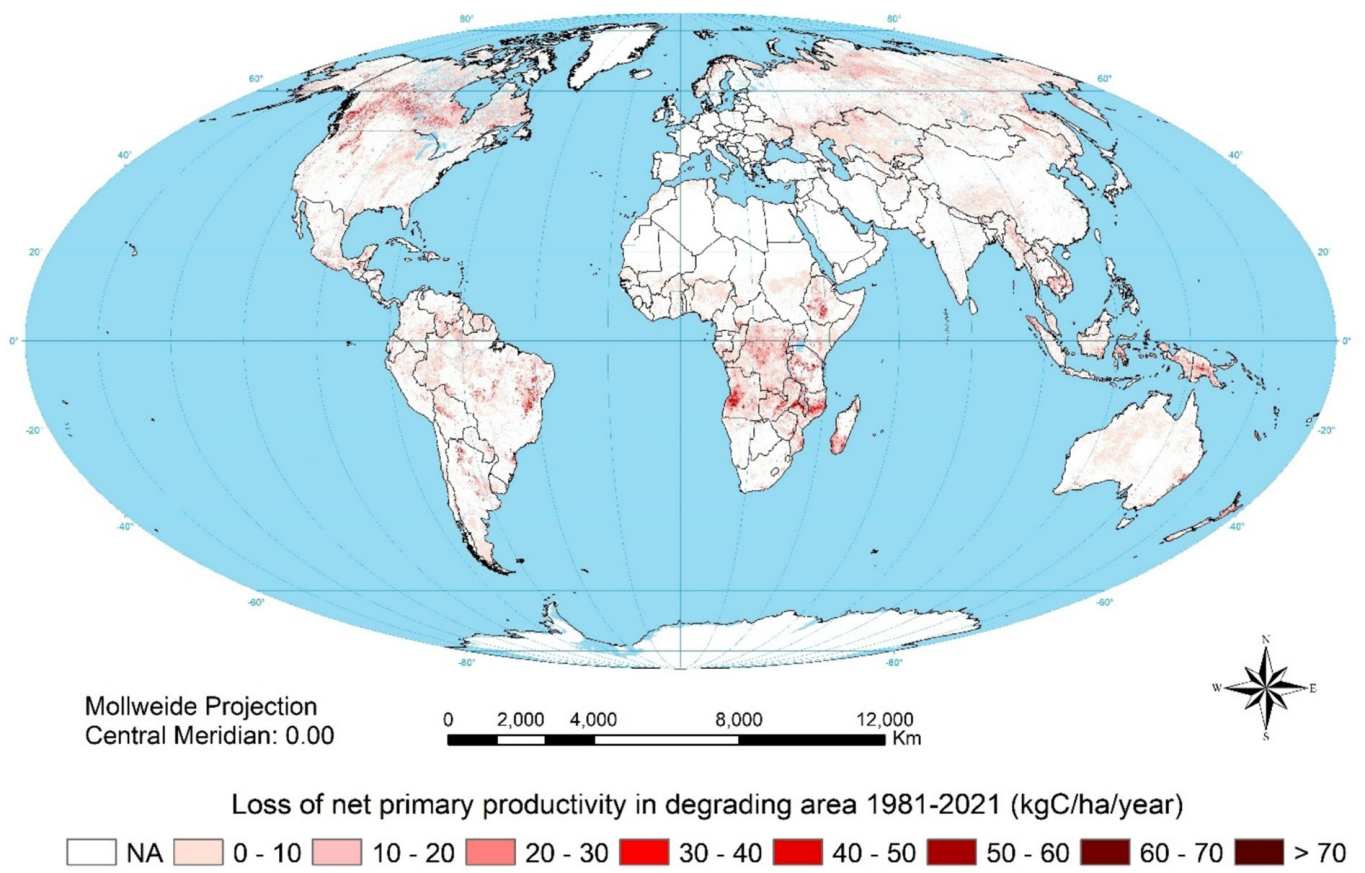
NPP loss in degrading areas 1981–2021

Figure 4 shows NPP loss in the degrading areas. Globally, annual loss of NPP over the period 1981–2021 is 40.7 million tonnes of carbon (MtC); somewhat less than the annual loss of 41.5 MtC during 1981–2003. Overall, the degrading area has been increasing but the annual loss of NPP has declined.

Table 2 shows that the most extensive degrading areas are in Russia, Canada, USA, DR Congo, and Kazakhstan—not entirely according to the size of the countries: in terms of absolute NPP loss, the ranking is Russia, Canada, DR Congo, Kazakhstan, and China. Inevitably with their numerous inhabitants, China and India head the list of affected population with 119 and 110 million, respectively, but in both cases land degradation directly affects only 8% of their total population. In contrast, the 95 million people living on degrading land in Indonesia, 61 million in Ethiopia and 57 million in DR Congo comprise 34, 49 and 57% of their respective populations.

Comparing the two time periods (Table 3), there are big proportional increases in land degradation in Russia, Canada, USA, DR Congo, and Kazakhstan while there are big decreases in China, Indonesia, and Australia. The numbers of people involved reveal both triumphs and disasters. In China, the number of people directly affected fell from 437 to 119 million, in India from 177 to 110 million, and in Bangladesh from 73 to 5 million. In each case, millions have moved to the cities; by one estimate, China’s rural population decreased by 241 million between 1995 and 2014 (Kundu et al. 2020) but there has also been internal migration to the cities in Nigeria where the number of people in degrading areas still rose from 17 to 60 million, in DR Congo from 32 to 57 million, in Uganda from 4 to 25 million, and in Afghanistan from 0.7 to 8.8 million. And in scores of low- and middle-income countries, land degradation went from bad to worse without much effect on the global aggregate but with a devastating toll on their people and societies.

### Land improvement

In contrast, 26.2% of global land has improved over 1981–2021, 10.5% more than over 1981–2003. By area, Russia leads with 4.9 million km^2^, followed by Australia with 3.6 million km^2^ and China with 3.4 million km^2^. But the proportions of improved land in these huge countries are otherwise: Russia 29%, Australia 48%, China 36% and, by this measure, Portugal is preeminent its 83 thousand km^2^ of improved land constitutes 89% of the country. Comparing improving areas with global land cover (ESA Copernicus 2023), Table 5 shows that 31% is scrub, 24% is broadleaved forest, 18% is needle-leaved forest, 14% is cropland, and 11% is grassland. By proportion of each land cover type, improved land constitutes 27% of all scrublands, 31% of broadleaved forest, 32% of needle-leaved forest, 36% of cropland, and 24% of grassland.

**Fig. 5:**
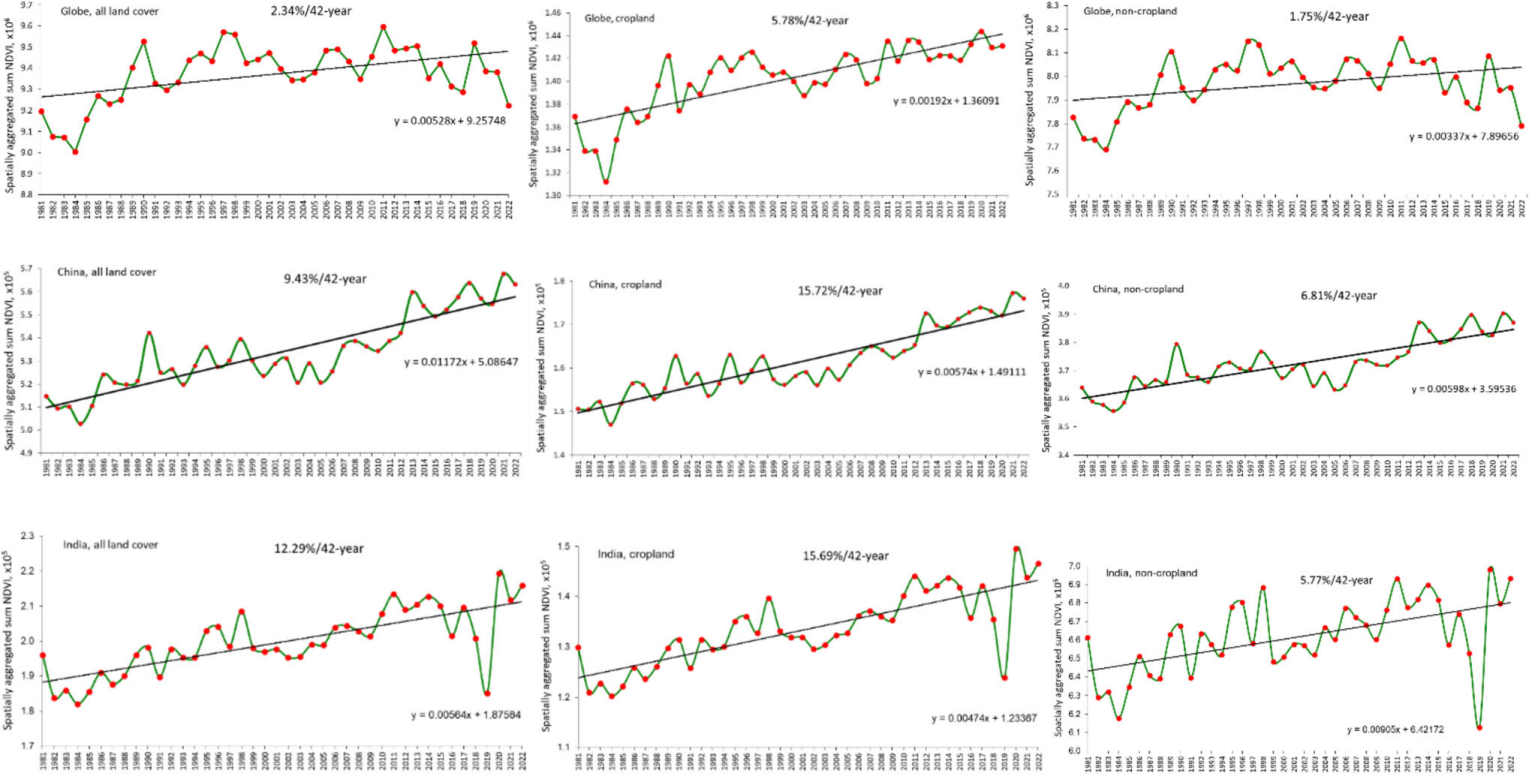
Aggregate NDVI, 1981–2022 for the World, China and India for all land cover, cropland and non-cropland

Figure 3 and Table 4 highlight dramatic recent improvements in cropland in China, India, and the European Union that confirm optimism about the potential of consistent, effective policies. Figure 5 depicts the global trend in the annual sum NDVI between 1981 and 2021, also distinguishing cropland and non-cropland and, individually, China and India. Globally, annual sum NDVI increased by 2.3% (*P* < 0.05), 5.8% in cropland and 1.8% in non-cropland, but both China and India exceeded the global trends threefold, most notably in their improving cropland.

Since the 3rd Plenum of the 11th Central Committee of the Chinese Communist Party in 1978, policy has promoted transformation of the peasantry to a ‘new type of professional farmer’, supported by the world’s largest public agricultural extension service (Babu et al. 2015). At the same time, between 1978 and 2022, the proportion of China’s population living in cities rose from 17.9 to 61.4%. In the first decade of the new millennium, the built-up area rose by 78.5% (Bai et al. 2014), half of it by building directly on arable land and, yet, the sown area increased from 146 million ha in 1980 to 166 million ha in 2019 and gross production doubled (NSBC 2020a, 2020b). In India, agricultural input subsidies and price support have been critical. The European Union has operated a common agricultural policy since 1962; its focus has changed over the years but support for farmers has been unstinting. Australian farmers are not subsidised but they apply well-informed common sense; in recent years, nearly all of them have adopted no-till Conservation Agriculture, otherwise known as *regenerative agriculture* (Dent and Boincean 2023; Kassam and Kassam 2023)—forsaking the plough; maintaining ground cover by cash crops and cover crops and, between crops, a mulch of crop residues; and planting directly through the protective surface mulch. Australians have also learned not to allow accumulation of tinder on their forest floors, reverting to the previous successful practice of controlled burning. Countries with unacceptable levels of land degradation must surely consider whether any of these avenues of improvement is open to them. The Australian way has the advantage of requiring minimal government intervention—it has been adopted across 15% of the world’s cropland as a farmers’ movement (Kassam et al. 2023).

**Fig. 6:**
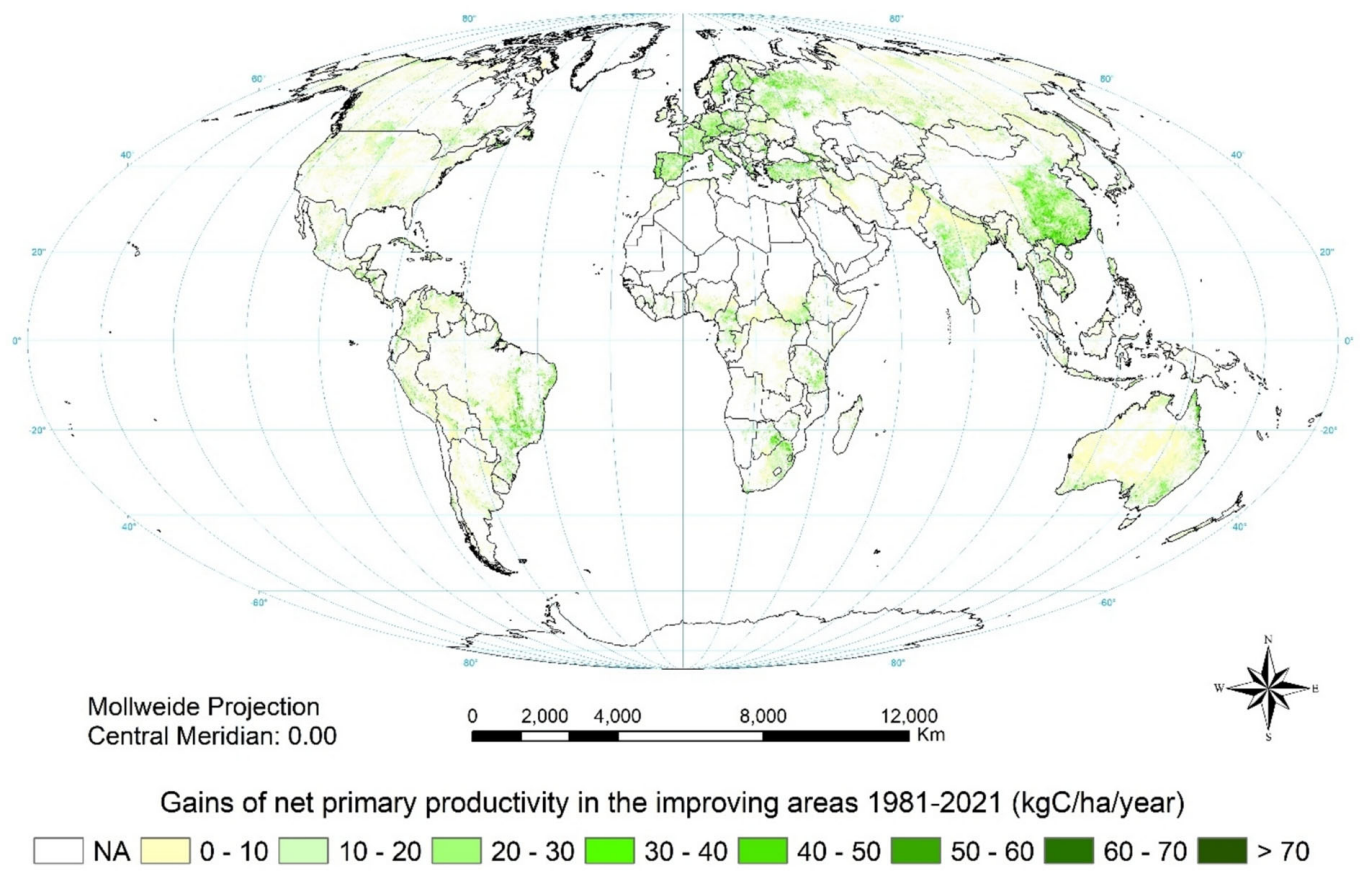
Global NPP gains in the improving areas over 1981–2021

Only one quarter of improving areas showed improvement throughout both periods (Fig. S2). Figure 6 shows NPP gain in improving areas over the whole 41 years: globally, the annual NPP gain is 57.8 MtC, significantly greater than the 1981–2003 figure, leading to a total NPP gain of 2.36 GtC.

If we take the net NPP gain or loss as an indicator of SDG15 *land degradation neutrality,* then Canada falls farthest short with a net NPP loss of 237.3 MtC. There were further big losses in DR Congo, Kazakhstan and Angola, amounting to 59, 47 and 41 MtC, respectively (Table 2). The greatest net gains have been in China, Australia, and Russia at 286, 123 and 96 MtC, respectively. However, when treated as a single entity, the European Union achieves the largest net gain at 300 MtC with no individual member state showing a net loss.

The improving areas more than doubled from 2003 to 2021 (Table 4). Many more people now live in improving areas—2.9 billion in 2021 compared with 0.8 billion in 2003: 664 million more people live on improving land in China, 538 million more in India, 118 million more in Bangladesh, 58 million more in Vietnam, and 20 million more in Nepal.

### Interpretation of NDVI/NPP; limitations and policy considerations

Serendipitously, weather satellites hold up a mirror to biological productivity just as much as to weather systems. While these data illustrate interesting vegetation dynamics, it is worth reiterating that changes in climate-adjusted NDVI/NPP serve only as a proxy for land degradation or improvement. In particular, ambiguous data from the boreal forest belt almost certainly reflect catastrophic forest fires, as recorded by the special fire channel on MODIS Terra and Aqua satellites (Szpakowski and Jensen 2019; CAS 2023; You 2023), and outbreaks of pests like the mountain pine beetle (Kurz et al. 2008). These are part of the natural cycle. Therefore, we might expect recovery but ecosystem recovery is slow in cold and dry regions and if, *as seems likely*, these events are themselves related to climate change, the ecosystem may not recover. Moreover, changes in land use from forest to cropland, increases in grazing pressure, or market adjustments to a less-intensive management will all decrease NDVI. These changes might or might not be accompanied by soil erosion, salinity, or other symptoms of land degradation that require acute attention. In the same vein, pastoralists will not consider bush encroachment as land improvement although it may increase biomass.

So, how is Gaia doing? Over the period of study, gains have generally outpaced losses: global biological productivity has been increasing, and increasing faster in the last 20 years than in the twenty years before—benefitting from increasing atmospheric carbon dioxide concentration, nitrogen deposition, temperature, and rainfall. This statement requires qualification. First, Fig. 3 illustrates significant regional disparities: between improvements in the European Union, China and India; and big losses across boreal forests, sub-Saharan Africa, parts of southeast Asia, the Steppes, Cerrado, Pampas, and Chaco. The *green revolution* involved unprecedented application of fertilisers, irrigation, and new crop varieties that can respond to these inputs by economies that could afford them: people that could not afford them have been bypassed. Secondly, crude global and national accounting conceals significant land degradation. The patterns of megafires in boreal forests and degradation of globally significant grain-producing areas on the steppes were already evident in the 1981–2003 data. They are more prominent in the extended data, which may be attributed to enhanced global heating and accompanying drought in continental areas where rainfall has not also increased. Crop yields across the steppes have stalled or even decreased during the study period; air temperatures have been increasing by 0.45 °C every decade but precipitation has remained much the same so, *e.g.* in southern Ukraine the soil water deficit has increased from 300 mm in 1990 to 400 mm in 2000 and is heading for 550 mm in 2050 and 700 mm in 2100. This trend will render half of Ukraine’s current arable land unsuitable for rainfed crops by 2050 and two-thirds by 2100 (Romaschenko et al. 2025). Thirdly, more primary productivity is not necessarily better and, certainly, not sustainable if is achieved by sacrificing biodiversity, expending more energy that it produces, and depleting aquifers. Regenerative agriculture offers equally productive but more sustainable options.

Gaia’s gains in carbon capture are dwarfed by man-made carbon emissions: between 1981 and 2021, the land has captured 694 MtC (Table 2), merely a few per cent of 10 Gt annual fossil carbon emissions. Moreover, the increasing extent and frequency of megafires and unremitting forest clearance expose the fragility of the grand strategy embraced by the Kyoto Protocol of the UN Convention on Climate Change that relies on forests as a carbon sink. We should do well to leave fossil fuels in the ground and invest in carbon storage with a longer half-life, such as soil organic matter that holds more carbon than all standing vegetation and the atmosphere combined.

The global assessment and trends over policy-significant 20-year periods show that 1.2 billion people live in areas that have degraded over the past 20 years, compared with 2.9 billion people living in areas that have improved. This underscores the power of consistent, targeted policies on land resilience and sustainability but, remembering Gaia’s greater domain, the 70% that is ocean does not figure in our calculations. Nor does the increasing area occupied by urban and industrial development and infrastructure. Both cry out for new lines of research. Urban greenness attracts increasing attention in the field of public health and well-being, particularly the air-conditioning roles of vegetation, trapping poisonous pollutants and dramatically lowering summer temperatures. GIMMS data at 9-km definition are too coarse to be useful in this case but MODIS data might be employed and cities have the resources to initiate specific, street-by-street programmes using drones.

For all the caveats, NPP data are of immediate practical value to policy makers who need to know exactly where and to what extent biologically significant changes are happening. Long-term trends of NDVI/NPP derivatives provide a globally consistent yardstick. They direct attention to places that demand investigation and action on the ground, as intended under the parent programme of the UN Food and Agriculture Organisation: Land Degradation Assessment in Drylands (Biancalani et al. 2013). It is hard to distinguish between statistically significant changes and practically significant changes—short-term variability reflects seasonal weather and local management decisions—but maps of long-standing trends should be on the desks, or pinned to the wall, in front of every policy maker. They are prescient predictors of political trouble.

## Conclusions

Changes in land degradation and improvement since 1981 have been assessed by proxy using remotely sensed NDVI translated into net primary productivity. This presents a different picture from previous qualitative assessments of land degradation that compounded current and historical processes but NDVI/NPP is only a proxy—some elements of land degradation and improvement, such as biodiversity, are not captured by NPP.

In the period 1981–2021, 28.5% of land was degrading—most notably through megafires in boreal forests, land clearance and cultivation in sub-Saharan Africa and the East Indies, and bare ground across the steppes. The degraded area was 4.5% greater than in 1981–2003 but fewer people were affected—1.2 billion compared to 1.5 billion. Over the longer period, consistent policies on sustainability increased biological productivity on 26% of land (10.5% more than 1981–2003).

At the present time, political and financial support for continuation of these policies is uncertain. But evidence-based decision-making still needs evidence. Developing any new policies will require quantitative data on the extent and degree of active land degradation to show where action is necessary.

Every line of Tables 3 and 4 tells a story but GIMMS holds more, and more-detailed information; significant variations within countries can be distinguished by regional analysis and MODIS NPP can deliver this information at the field scale.

It remains up to politicians to ask the right questions: Where, exactly, is productivity better, or worse? And why? That is to say: What are the proximal and underlying causes? What can and should be done to solve the problems or take advantage of the opportunities—in the short term and in the longer term? Who is responsible? Is it me? And to act upon the answers. Clearly, information is needed for the appropriate recommendation domains which, in most cases, will be nested within the national pictures that we have presented; and at the landscape level, the defining roles of terrain, soils and drainage, strategy, and implementation can be incorporated.

## Supplementary Information

Below is the link to the electronic supplementary material.

Supplementary file1 (PDF 369 KB)
