# Supplementary material for: How is Gaia doing? Trends in global land degradation and improvement
Source: Ambio. 2025 Apr 24;54(11):1902–38. doi: 10.1007/s13280-025-02179-9 (PMC12480325; doi:10.1007/s13280-025-02179-9)
Supplement: Supplementary file 1 — Supplementary file1 (PDF 369 KB) [file 13280_2025_2179_MOESM1_ESM.pdf]

Ambio

Supplementary Information
This supplementary information has not been peer reviewed.

Title: **How is Gaia doing? Trends in global land degradation and improvement**

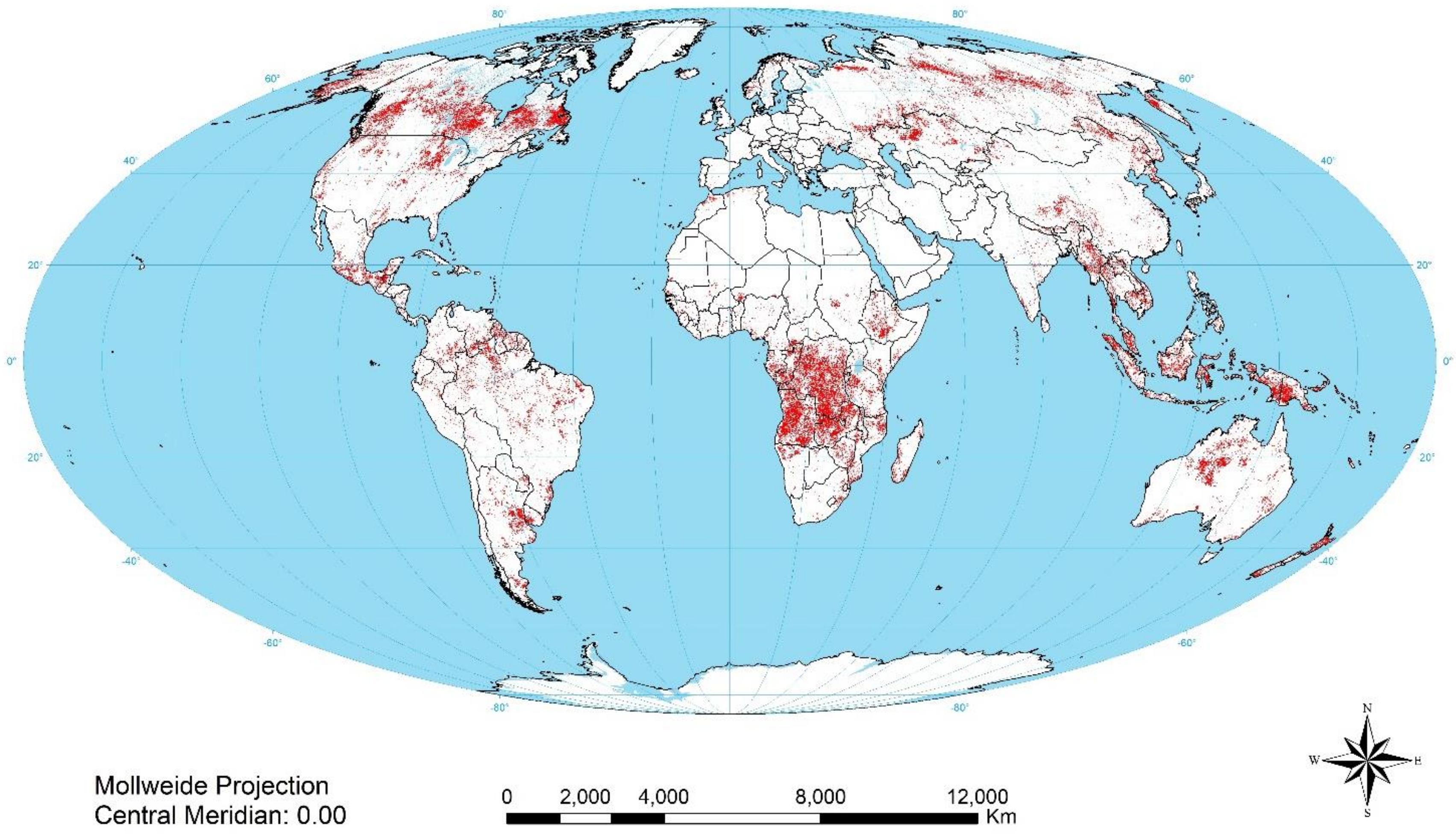

Fig. S1 Continued land degradation throughout 1981-2003 and 1981-2021

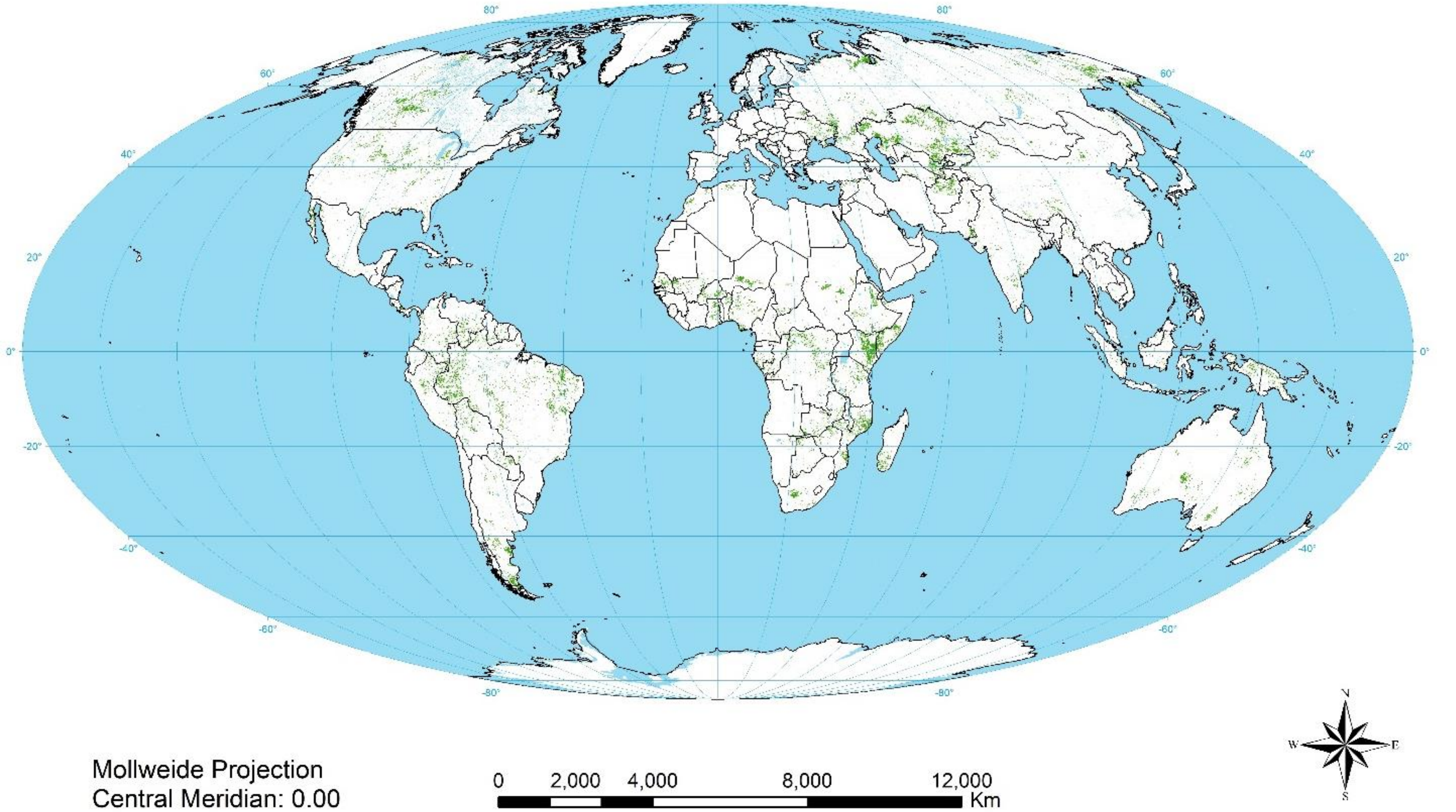

Fig. S2 Continued land improvement throughout 1981-2003 and 1981-2021
